# Experimental Arthritis Inhibits Adult Hippocampal Neurogenesis in Mice

**DOI:** 10.3390/cells11050791

**Published:** 2022-02-24

**Authors:** Kitti Rusznák, Ádám István Horváth, Kinga Pohli-Tóth, Anett Futácsi, Ágnes Kemény, Gabriella Kiss, Zsuzsanna Helyes, Boldizsár Czéh

**Affiliations:** 1 Structural Neurobiology Research Group, Szentágothai Research Centre, University of Pécs, 7624 Pécs, Hungary; rusznak.kitty@gmail.com (K.R.); 1998tothkinga@gmail.com (K.P.-T.); futacsi.anett@gmail.com (A.F.); 2 Department of Laboratory Medicine, Medical School, University of Pécs, 7624 Pécs, Hungary; kiss.gabriella2@pte.hu; 3 Molecular Pharmacology Research Group, Szentágothai Research Centre, University of Pécs, 7624 Pécs, Hungary; adam.horvath@aok.pte.hu (Á.I.H.); zsuzsanna.helyes@aok.pte.hu (Z.H.); 4 Department of Pharmacology and Pharmacotherapy, Medical School, University of Pécs, 7624 Pécs, Hungary; kemeny.agnes@pte.hu; 5 Department of Medical Biology, Medical School, University of Pécs, 7624 Pécs, Hungary; 6 PharmInVivo Ltd., 7624 Pécs, Hungary; 7 Histology and Light Microscopy Core Facility, Szentágothai Research Centre, University of Pécs, 7624 Pécs, Hungary

**Keywords:** animal model, cell proliferation, chronic inflammation, cytokine, chemokine, chronic pain, dentate gyrus, hippocampus, microglia, neuroplasticity

## Abstract

**Background:** Adult-born neurons of the hippocampal dentate gyrus play a role in specific forms of learning, and disturbed neurogenesis seems to contribute to the development of neuropsychiatric disorders, such as major depression. Neuroinflammation inhibits adult neurogenesis, but the effect of peripheral inflammation on this form of neuroplasticity is ambiguous. **Objective**: Our aim was to investigate the influence of acute and chronic experimental arthritis on adult hippocampal neurogenesis and to elucidate putative regulatory mechanisms. **Methods**: Arthritis was triggered by subcutaneous injection of complete Freund’s adjuvant (CFA) into the hind paws of adult male mice. The animals were killed either seven days (acute inflammation) or 21 days (chronic inflammation) after the CFA injection. Behavioral tests were used to demonstrate arthritis-related hypersensitivity to painful stimuli. We used in vivo bioluminescence imaging to verify local inflammation. The systemic inflammatory response was assessed by complete blood cell counts and by measurement of the cytokine/chemokine concentrations of TNF-α, IL-1α, IL-4, IL-6, IL-10, KC and MIP-2 in the inflamed hind limbs, peripheral blood and hippocampus to characterize the inflammatory responses in the periphery and in the brain. In the hippocampal dentate gyrus, the total number of newborn neurons was determined with quantitative immunohistochemistry visualizing BrdU- and doublecortin-positive cells. Microglial activation in the dentate gyrus was determined by quantifying the density of Iba1- and CD68-positive cells. **Results**: Both acute and chronic arthritis resulted in paw edema, mechanical and thermal hyperalgesia. We found phagocytic infiltration and increased levels of TNF-α, IL-4, IL-6, KC and MIP-2 in the inflamed hind paws. Circulating neutrophil granulocytes and IL-6 levels increased in the blood solely during the acute phase. In the dentate gyrus, chronic arthritis reduced the number of doublecortin-positive cells, and we found increased density of CD68-positive macrophages/microglia in both the acute and chronic phases. Cytokine levels, however, were not altered in the hippocampus. **Conclusions**: Our data suggest that acute peripheral inflammation initiates a cascade of molecular and cellular changes that eventually leads to reduced adult hippocampal neurogenesis, which was detectable only in the chronic inflammatory phase.

## 1. Introduction

Neurogenesis in the adult brain is a unique form of neuroplasticity. Generation of newborn neurons has been reported in the adult hippocampus of humans [1,2], non-human primates [3,4] and rodents [5,6]. Newly generated neurons of the hippocampal dentate gyrus seem to play a significant role in specific forms of learning and memory processes [7,8,9,10,11]. Furthermore, disruption of adult neurogenesis may contribute to the development of various neuropsychiatric disorders, including depressive disorders [12,13]. 

Numerous external and internal factors influence the production of newborn neurons. An inflammatory niche and circulating pro-inflammatory cytokines are well-known inhibitors of adult neurogenesis [14,15,16,17,18,19,20]. Neuroinflammation is a potent inhibitor of adult hippocampal neurogenesis [21,22,23,24], but the available data regarding the influence of peripheral or systemic inflammation on this process are controversial [25]. The first studies investigating the effects of peripheral inflammation injected a bacterial endotoxin (lipopolysaccharide: LPS) to induce systemic inflammation in mice and investigated its long-term consequences on neurodegeneration, hippocampal neurogenesis and cognitive functions [26,27,28,29]. However, relatively few studies, thus far, have applied a model with inflammation of a specific organ. Some of these studies administered dextran sulfate sodium (DSS) to elicit experimental colitis and investigated its influence on adult neurogenesis [30,31,32]. The first study reported that both acute and chronic colitis can inhibit the production of adult-born neurons in the dentate gyrus and increase the expression of the activated microglia marker Iba1-protein in the hippocampus [30]. This was confirmed later by a demonstration in which DSS-treated mice had reduced adult hippocampal neurogenesis and increased activation of microglia and astrocytes [31]. More recently, a comprehensive study reported that acute colitis can in fact increase neurogenesis; though animals with chronic colitis had normal neurogenesis, the newborn neurons displayed deficits during their integration into the functional circuitry [32].

Another research line applied complete Freund’s adjuvant (CFA) injections to trigger rheumatoid arthritis, which resulted in a transient increase in hippocampal precursor cell proliferation and neurogenesis in female mice [33]. More recently, the effect of chronic peripheral inflammation on neuroplastic changes in different regions of the central nervous system (CNS) has been studied thoroughly in a TNF-α transgenic mouse model of rheumatoid arthritis [34,35]. These transgenic mice manifest severe erosive arthritis with increased IL-1β and IL-6 expression in their joints together with highly elevated TNF-α levels in the serum, but none of these alterations could influence adult hippocampal neurogenesis, anxiety levels, or depressive-like behavior of the animals [34]. Other studies, using unilateral CFA-treatment to create chronic pain models, also reported a reduced rate of adult neurogenesis [36,37]. Notably, depression is highly prevalent among patients with rheumatoid arthritis (or chronic pain), and it is also associated with poorer clinical outcomes [38,39]. Finally, a very recent publication demonstrated that adult hippocampal neurogenesis is involved in the maintenance of pathological pain induced by peripheral neuropathy [40].

Due to these contradictory findings in the literature, our aim was to further characterize the influence of peripheral inflammation on adult hippocampal neurogenesis using the CFA-induced arthritis model. We injected CFA to the hind limbs of male mice to trigger acute and chronic arthritis, and then we characterized the animals behaviorally. Next, inflammatory responses were studied with cellular and molecular methods, and, finally, we quantified adult-born neurons in the hippocampus. One subset of animals was killed seven days (acute inflammation) after the CFA injection, while another set was killed after 21 days (chronic inflammation). We used behavioral tests to assess the arthritis-related hypersensitivity to painful stimuli. Bioluminescence imaging was applied to verify local inflammation, and blood cell counts were performed to examine the systemic inflammatory response. We measured cytokine/chemokine levels of TNF-α, IL-1α, IL-4, IL-6, IL-10, KC and MIP-2 in the hind paws, peripheral blood and hippocampus. Cytogenesis and newborn neurons of the dentate gyrus were visualized and quantified using BrdU- and doublecortin-immunohistochemistry. Activated microglia and macrophages were labelled with Iba1- and CD68-antibodies, and cell densities were determined in the dentate gyrus. Our hypothesis was that chronic peripheral inflammation should elicit a parallel neuroinflammatory response in the hippocampus, and thereby inhibit adult neurogenesis in the dentate gyrus.

## 2. Materials and Methods

### 2.1. Animals

Experiments were performed on adult (9–12 week-old) male C57BL/6J mice weighing 20–30 g. In total, 80 mice were used in this experiment. They were bred in the Laboratory Animal House of the Department of Pharmacology and Pharmacotherapy at the University of Pécs and kept (group-housed) in the Laboratory Animal House of the Szentágothai Research Centre under a standard 12-h light/dark cycle at 24 ± 2 °C with a relative humidity of 50–60%. Food and water were available *ad libitum* in the home cages.

This study was carried out according to European legislation (Directive 2010/63/EU) and Hungarian Government regulation (40/2013., II. 14.) regarding the protection of animals used for scientific purposes. The experiments were approved by the Animal Welfare Committee of the University of Pécs and the National Scientific Ethical Committee on Animal Experimentation of Hungary and licensed by the Government Office of Baranya County (license number: BA02/2000-28/2020).

### 2.2. Experimental Design

**Figure 1:**
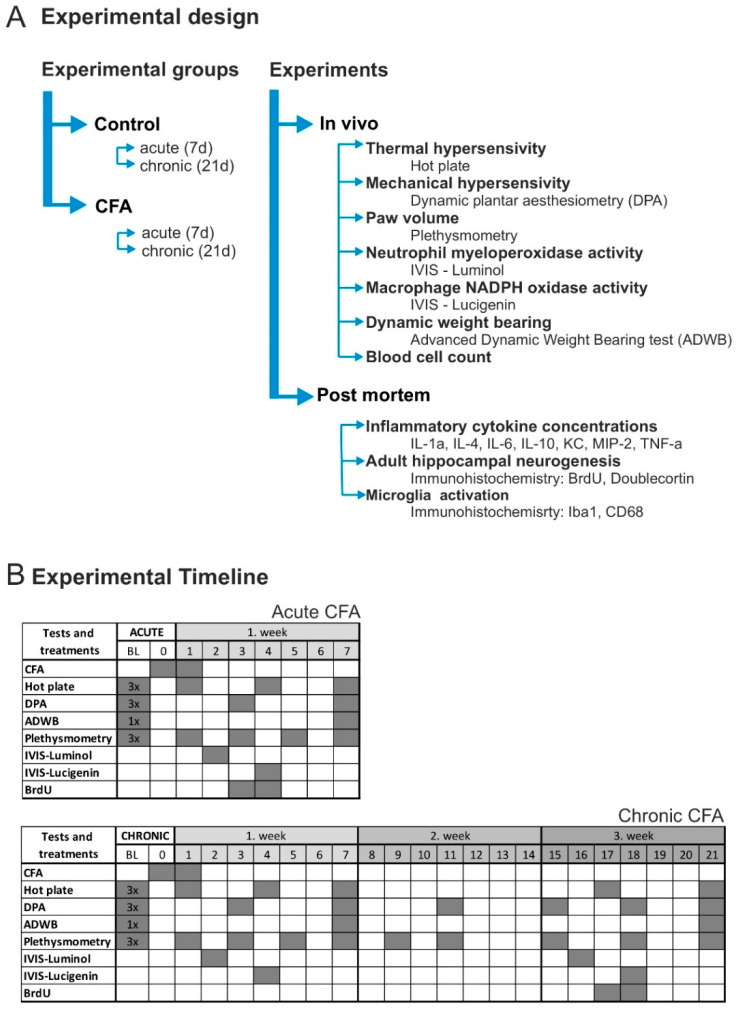
Experimental design and experimental timeline. (**A**) Mice were randomly divided into four groups: Acute Control; Chronic Control; Acute CFA and Chronic CFA (*n* = 20/group). Various behavioral, physiological and histopathological studies were carried out to characterize the consequences of acute and chronic CFA-induced arthritis. (**B**) The experimental schedules for the Acute CFA- and Chronic CFA-treated animals, as well as their corresponding controls. Behavioral tests, in vivo imaging and treatments were performed on those days which have gray background. Abbreviations: advanced dynamic weight bearing (ADWB); baseline (BL); complete Freund’s adjuvant (CFA); dynamic plantar aesthesiometry (DPA).

The experimental design, including the animal groups, behavioral tests and physiological assessments, as well as the timeline of the procedures, is depicted in Figure 1. First, the animals were allowed to habituate to the new housing conditions for 14 days. Behavioral baseline values were determined during the second week of this habituation period. Afterwards, the animals were randomly divided into four experimental groups according to the treatment protocols and survival time: (1) Acute Control; (2) Acute CFA; (3) Chronic Control; (4) Chronic CFA (*n* = 20/group). Mice were killed either 7 days (acute phase) or 21 days (chronic phase) after the CFA injections. All behavioral tests were performed during the light phase of the animals. We tested mice in an alternating order from the different experimental groups using a predefined sequence to minimize the potential effect of the circadian rhythm on the behavioral performance. One set of mice was perfused with a fixative and processed for histopathology, while the other set was killed for collection of blood and tissue samples of the hind paws and hippocampi to determine cytokine levels.

### 2.3. Induction of Joint Inflammation

CFA-induced arthritis is a well-established rodent model of chronic arthritis [41,42,43,44], and it was elicited according to our standard protocol [45,46]. Inflammation of the right tibiotarsal joint was induced by subcutaneous injection of 20 µL of complete Freund’s adjuvant (CFA; consisting of heat-killed Mycobacterium tuberculosis suspended in paraffin oil, 1 mg/mL; Sigma Aldrich, St. Louis, MO, USA) into the plantar surface of the right hind paw and to the tail root under i.p. ketamine (100 mg/kg Calypsol, Gedeon Richter Plc., Budapest, Hungary) and xylazine (5 mg/kg Sedaxylan, Eurovet Animal Health B.V., Bladel, The Netherlands) anesthesia (on Day 0). An additional tail root injection was administered in the same volume on Day 1 to boost the systemic inflammatory effect.

### 2.4. Mechanonociception

Mechanical hypersensitivity was examined by measuring the mechanical paw withdrawal threshold of the hind paws using a dynamic plantar aesthesiometer (DPA, Ugo Basile, Gemonio, Italy). Mechanical hyperalgesia was calculated as the percentage decrease in the mechanonociceptive thresholds compared to the pre-treatment values [45,46]. Mice were placed in plexiglas boxes with a steel mesh floor, and a force of 2.5 g/s was applied via a 0.5 mm diameter straight metal filament to the plantar surface of the hind paw until the animal lifted its foot. The mechanical withdrawal nociceptive threshold, inducing paw withdrawal, was the pressure expressed in grams (g). Animal numbers in this test: Acute Control (*n* = 11); Acute CFA (*n* = 11); Chronic Control (*n* = 15); Chronic CFA (*n* = 15).

### 2.5. Thermonociception

Thermal hypersensitivity was documented by the decreased latency in nocifensive behavior in response to noxious heat stimuli. The latency of nocifensive behavior (lifting, shaking or licking either hind paw) was measured using the Hot/Cold Plate analgesia meter (Ugo Basile, Gemonio, Italy). Latency time was expressed in seconds. The surface of the plate was maintained at 50 °C, and the cut-off time was 20 s. Animal numbers in this test: Acute Control (*n* = 11); Acute CFA (*n* = 11); Chronic Control (*n* = 15); Chronic CFA (*n* = 15).

### 2.6. Bodyweight Imbalance

Dynamic bodyweight imbalance (incapacitance) was determined with an advanced dynamic weight bearing apparatus (Bioseb, Vitrolles, France) and expressed as the ratio of the weight distributed on one hind limb divided by the total weight borne on both hind limbs [(weight borne on the left or right hind limb)/(weight borne on both hind limbs)] × 100 [47]. Animal numbers: Acute CFA (*n* = 19); Chronic CFA (*n* = 15).

### 2.7. Hind Paw Volume

Hind paw volumes were measured with a Plethysmometer (Ugo Basile, Gemonio, Italy) and expressed in cm^3^. Animal numbers: Acute Control (*n* = 11); Acute CFA (*n* = 11); Chronic Control (*n* = 15); Chronic CFA (*n* = 15).

### 2.8. In Vivo Bioluminescence Imaging

Inflammatory reaction of the joints was examined by in vivo bioluminescence imaging. We measured neutrophil myeloperoxidase (MPO) activity with luminol (5-amino-2,3-dihydro-1,4-phthalazine-dione) and macrophage NADPH oxidase (Phox) activity with lucigenin (bis-*N*-methylacridinium nitrate) in order to detect reactive oxygen species [48]. Imaging of the animals was performed 10 min after the i.p. injection of luminol sodium salt (150 mg/kg; Gold Biotechnology, Olivette, MO, USA) dissolved in phosphate buffered saline (PBS; 30 mg/mL) and lucigenin (25 mg/kg; Tokyo Chemical Industry Co., Ltd., Tokyo, Japan) dissolved in saline (2.5 mg/mL) using the IVIS Lumina III imaging system (PerkinElmer, Waltham, MA, USA). Acquisition times were 120 s for luminol-derived bioluminescence and 180 s for lucigenin-derived bioluminescence (Binning = 8, F/Stop = 1). Identical regions of interest (ROIs) were applied on the hind paws and ankle joints, and luminescence was expressed as the total radiance (total photon flux/s) within the ROIs [49]. Animal numbers: Acute Control (*n* = 24); Acute CFA (*n* = 21); Chronic Control (*n* = 9–10); Chronic CFA (*n* = 10).

### 2.9. Complete Blood Cell Count

Shortly before killing the mice, blood samples were collected in 500 µL Microtainer blood sampling tubes containing K_2_EDTA (Becton Dickinson, Hungary). Complete blood cell count was determined by a Sysmex XN-V automated hematology analyzer for veterinary use in a pre-defined mouse species whole blood mode. Animal numbers: Acute Control (*n* = 5); Acute CFA (*n* = 5); Chronic Control (*n* = 8); Chronic CFA (*n* = 8).

### 2.10. Cytokine Concentrations

Cytokine levels were determined postmortem from tissue samples of the ankles, peripheral blood and hippocampi. Blood samples were collected after decapitation. Excised and snap-frozen tissues were homogenized in cold PBS containing 10 mg/mL of phenylmethanesulfonyl fluoride (PMSF, Sigma Aldrich, P7626) on ice with IKA’s ultra-turrax device. Afterwards, samples were centrifuged for 20 min (4 °C, 4000 rpm), and supernatants were pooled from animals of the same treatment groups and stored at −80 °C.

A Milliplex assay based on the Luminex xMAP technology was performed to determine the protein concentrations of seven distinct cytokines/chemokines using customized Milliplex Mouse Cytokin/Chemokine Magnetic Bead Panel (Merck Millipore MCYTOMAG-70K, Merck Life Science Ltd., Budapest, Hungary). We measured concentrations of the following cytokines/chemokines: interleukin-1-alpha (IL-1α); interleukin-4 (IL-4); interleukin-6 (IL-6); interleukin-10 (IL-10); tumor necrosis factor alpha (TNF-α); keratinocyte-derived chemokine (KC) and macrophage inflammatory protein-2 (MIP-2). Following previous optimizations, all samples were tested undiluted in a blind-fashion and in duplicate. The experiment was performed according to the manufacturer’s instructions. Briefly, a 25 µL volume of each sample, control, and standard was added to a 96-well plate (provided with the kit) containing 25 µL of capture antibody coated, fluorescent color-coded bead mixture. Biotinylated detection antibodies and streptavidin-PE were added to the plate after the appropriate washing and incubation periods. After the last washing step, beads were resuspended in a 150 µL volume of drive fluid, and the plate was read on the Luminex MagPix instrument. The Five-PL regression curve was used to plot the standard curves for all analytes via analyst software that analyzed the bead classificator and reporter median fluorescence intensities. Cytokine concentrations were normalized to the protein concentrations of the tissue homogenates, and the results are reported as pg/mL wet tissue. Cytokine concentrations in blood samples are reported as pg/mg of wet tissue. Animal numbers: Acute Control (*n* = 6); Acute CFA (*n* = 6); Chronic Control (*n* = 6); Chronic CFA (*n* = 6).

### 2.11. BrdU Injection, Tissue Fixation and BrdU-Immunocytochemistry

The thymidine analog 5-bromo-2′-deoxyuridine (BrdU, Sigma-Aldrich, St. Louis, MO, USA) was used to label newly synthetized DNA in proliferating cells. BrdU was dissolved in sterile 0.9% saline (containing 0.007 N NaOH) at a concentration of 15 mg/mL. We injected BrdU (200 mg/kg, i.p.) into the animals on two consecutive days, 3–4 days before the perfusion (Figure 1B).

Mice were anesthetized deeply with a mixture of ketamine (Calypsol inj. 50 mg/mL, Richter Gedeon) and xylazine (Sedaxylan^®^ inj. 20 mg/mL, Eurovet Animal Health BV) 100/10 mg/kg and perfused transcardially with ice cold physiological saline and ice cold 4% paraformaldehyde in 0.1 M of phosphate buffer.

Brains were sectioned with a Leica VT1200 vibrating blade microtome (Leica Biosystems, BioMarker Ltd., Budapest, Hungary) to collect serial 50-µm coronal sections throughout the entire hippocampal formation along the septo-temporal axis. Every fourth section was slide-mounted on Superfrost slides (Menzel-Glaser, Braunschweig, Germany) and coded to ensure objectivity before processing for immunocytochemistry. BrdU-immunohistochemistry protocol was carried out according to our standard protocol [50]. Briefly, the key steps include: cellular DNA was denaturized in 0.1 M of citric acid at pH 6.0 and 95 °C for 20 min, and then sections were treated with 1% H_2_O_2_ in Tris for 20 min. Cellular membranes were permeabilized with 0.1% trypsin in 0.1 M of Tris for 10 min. Next, sections underwent acidification in 2N HCl in Tris for 30 min. Thorough rinsing in buffers was applied between each step. Nonspecific antibody binding was prevented by incubating sections for 1 h in 5% normal goat serum (NGS; Vector Laboratories, Burlingame, CA, USA). Subsequently, sections were incubated for one night at 4 °C with mouse anti-BrdU (1:5000, DAKO, Clone Bu20a, Catalog # M074401). After incubation with biotinylated goat anti-mouse IgG (1:200, Vector Laboratories) for 1 h at 4 °C, sections were incubated in avidin-biotin-horseradish peroxidase (1:500; Vectastaine Elite ABC Kit, Vector Laboratories) for 2 h at 4 °C. BrdU-labeled cells were visualized in 0.025% 3,3′-diaminobenzidine (DAB, Sigma-Aldrich) and 0.01% H_2_O_2_ in PBS for 10 min. After overnight drying at room temperature, sections were dehydrated in graded alcohol, cleared in xylene and coverslipped with Eukitt. Animal numbers used for all immunohistochemistry were: Acute Control (*n* = 8); Acute CFA (*n* = 8); Chronic Control (*n* = 9); Chronic CFA (*n* = 9).

### 2.12. Doublecortin (DCX), Ionized Calcium Binding Adaptor Molecule 1 (Iba1) and CD68 Immunocytochemistry

DCX-labeling was used to visualize immature neurons [51,52], Iba1-labeleing was applied to visualize activated microglia [53] and CD68- labeling was used to visualize activated microglia and macrophages [54]. Immunolabeling of DCX-positive cells was done as previously described in detail [50], and the protocols for Iba1- and CD68-immunohistochemistry were essentially the same. Briefly, free-floating serial sections were washed in 0.1 M of PBS, and then treated with 3% H_2_O_2_ for 30 min. After thorough rinsing, nonspecific binding was prevented by incubating the sections for 1 h in 3% normal goat serum (NGS; Vector Laboratories) in PBS containing 0.5% Triton X-100. Subsequently, sections were rinsed in PBS and incubated for one night at 4 °C with a rabbit anti-DCX antibody (1:3000, Cell Signaling Technology, Wien, Austria, Catalog # 4604), a polyclonal rabbit anti-Iba1 antibody (1:2000, FUJIFILM Wako Chemicals Europe GmbH, Neuss, Germany, Catalog # 019-19741) or with rabbit monoclonal anti-CD68 antibody (1:1000, AbCam, Cambridge, UK, Catalog # 283654). After repeated rinsing, sections were incubated with anti-rabbit biotinylated secondary antibody (1:200; Vector Laboratories) for 2 h, washed and incubated in avidin-biotin-horseradish peroxidase (1:200; Vectastaine Elite ABC Kit, Vector) for 2 h. Labelled cells were visualized with a DAB reaction (as above). Sections were mounted, dried and dehydrated in graded alcohol, and then cleared and coverslipped with Eukitt. All slides were coded before quantification to ensure objectivity. Images were acquired on a Nikon Eclipse Ti-U workstation (Auro-Science Consulting Ltd., Budapest, Hungary).

### 2.13. Cell Quantification in the Hippocampus

A single experimenter (KR), who was blind to the group identification of each animal, performed the data collection. The code was not broken until the cell counting analyses were completed. Cell counting was done using the Neurolucida (Version 7) reconstruction system (Microbrightfield, Colchester, VT, USA) attached to a Nikon Eclipse bright field microscope.

Cell quantification was carried out using a modified unbiased stereology protocol [55,56,57]. BrdU+, DCX+, Iba1+ and CD68+ cells were counted in a systematic manner in a complete series of 50-µm thick sections starting at a random position along the entire septo-temporal axis of the hippocampal formation (between –0.94 and –3.88 relative to Bregma [58]. Every fourth section was examined for each label, yielding 13–15 sections per animal. All BrdU+ and DCX-labeled cells in the granule cell layer, together with the subgranular zone, which is defined as a zone two cell bodies wide along the inner border of the granule cell layer, were counted regardless of size or shape. The Iba1-positive cells of the dentate gyrus were counted in an area that included the upper- and lower-blades of the granule cell layer and the hilus. The cells were examined under ×200 magnification, and cells in the outermost focal plane were omitted. The total number of BrdU+ and DCX+ cells was estimated by multiplying the number of cells counted in every fourth section by four. Both hemispheres were counted, and cell numbers are reported here as the total neuron numbers of both hemispheres. The Iba1- and CD68-positive cells were quantified in 7 sections/animal, and the results are expressed as densities (cell number/mm^3^).

### 2.14. Statistical Analysis

Results are expressed as mean ± SEM. Data analyses were performed using GraphPad Prism, Version 7. Behavioral data were analyzed with two-way repeated measures ANOVA or with two-way ANOVA (time × CFA treatment) followed by Sidak’s multiple comparisons post hoc test. The in vivo imaging data, blood cell counts, cytokine concentrations and cell numbers in the hippocampal dentate gyrus were analyzed with two-way ANOVA (time × CFA treatment) followed by Tukey’s multiple comparisons post hoc test. The level of significance was set at *p* < 0.05.

## 3. Results

### 3.1. CFA Treatment Induced Paw Edema, Thermal and Mechanical Hyperalgesia, as Well as Impaired Weight Distribution

**Figure 2:**
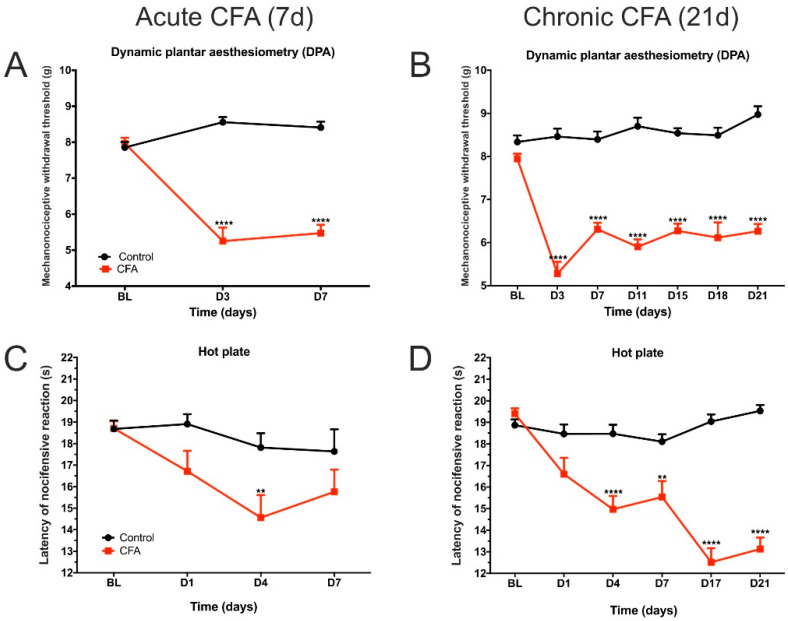

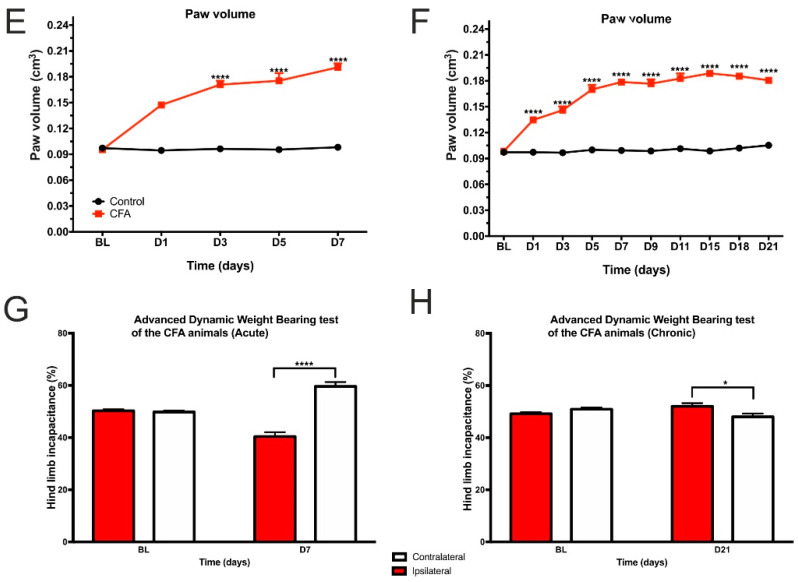
Behavioral testing revealed CFA-induced increase in pain sensitivity and edema of the inflamed hind limb. CFA-injected mice had reduced mechanonociceptive withdrawal threshold in acute (**A**) and chronic (**B**) inflammatory conditions, as revealed by the dynamic plantar aesthesiometry test. Mice with arthritis had increased thermal hypersensitivity in acute (**C**) and chronic (**D**) inflammatory conditions to noxious heat stimuli when tested with the hot plate test. CFA-treated animals developed sustained inflammatory edema both in acute (**E**) and chronic (**F**) conditions. Dynamic body weight distribution was also altered. Animals displayed bodyweight imbalance and reduced their bodyweight on the inflamed hind limbs during the acute phase (**G**), but this bodyweight imbalance was reversed on Day 21 in the Chronic CFA-treated animals (**H**). Y-axis of (**A**,**B**): mechanonociceptive withdrawal threshold. Y-axis of (**C**,**D**): latency of nocifensive reaction. Statistical analysis for A-F: two-way repeated measures ANOVA (time × CFA treatment) followed by Sidak’s multiple comparisons post hoc test. * *p* < 0.05; ** *p* < 0.01; **** *p* < 0.0001 *versus* control values at the same time point. Statistical analysis for G-H: two-way ANOVA (time × CFA treatment) followed by Tukey’s *post hoc* test. Abbreviations: baseline (BL); complete Freund’s adjuvant (CFA); day(s) (d and D). Red colored lines in B, D and F indicate the CFA-treated animals similarly to A, C, and E.

The CFA-injected animals developed behavioral symptoms indicating chronic pain in their inflamed hind limbs (Figure 2). They had hypersensitivity to mechanical stimuli from Day 3 onwards (Figure 2A,B). For a detailed statistical analysis of these data, see Supplementary Table S1. Similarly, the CFA-treated animals displayed thermal hypersensitivity to noxious heat stimuli (Figure 2C,D, statistics in Supplementary Table S2). The CFA-injected mice had persisting edema of the inflamed hind paws (Figure 2E,F, statistics in Supplementary Table S3). Furthermore, animals with arthritis displayed significant bodyweight imbalance during the acute inflammatory phase (Figure 2G, statistics in Supplementary Table S4), which was reversed in the chronic inflammatory phase (Figure 2H, statistics in Supplementary Table S5). Overall, these data clearly document that the CFA-treated animals were suffering from chronic pain.

### 3.2. CFA-Treatment Induced Local Inflammation in the Hind Limbs

**Figure 3:**
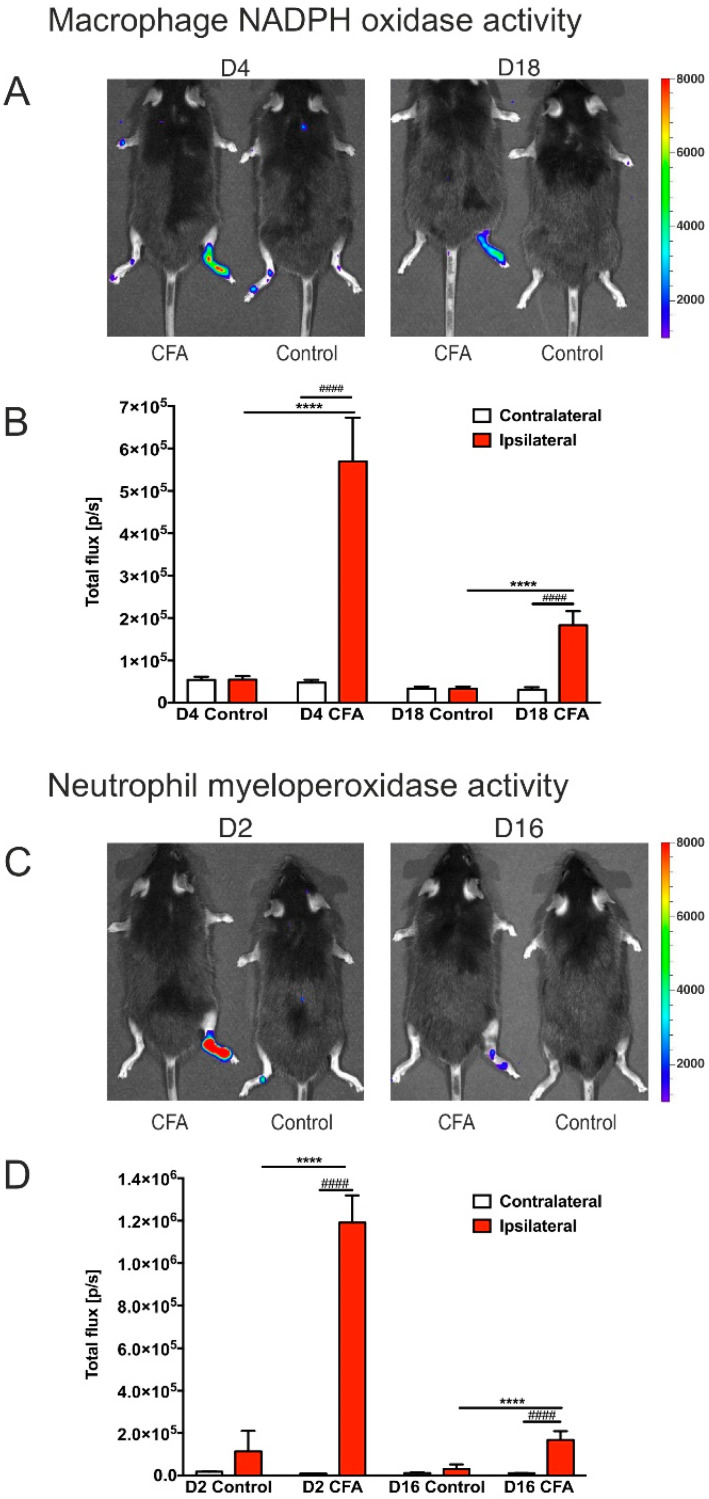
In vivo bioluminescence imaging demonstrating local inflammation, i.e., the presence of reactive oxygen species generated by the inflammatory phagocytes. (**A**) Images of CFA-injected and control mice. This method detects the macrophage NADPH oxidase activity after injection of lucigenin into the animals. Lucigenin reacts with the macrophage NADPH oxidases and gives a bioluminescence signal. Note the bioluminescence signals of the right hind limbs. (**B**) Quantitative data of the bioluminescent signals. (**C**) Bioluminescence signals were evoked by the injection of luminol, which reacts with the myeloperoxidase enzymes of the neutrophil granulocytes. (**D**) Quantitative data of the bioluminescence signals. The bioluminescence signal was significantly increased both in the acute and chronic phases of the inflammation. Statistical analysis: two-way ANOVA (time × CFA treatment) followed by Tukey’s multiple comparisons post hoc test. **** *p* < 0.0001 versus the ipsilateral hind limbs of the control mice at the same time points. #### *p* < 0.0001 versus the contralateral hind limbs of the CFA-injected animals at the same time points. Abbreviations: complete Freund’s adjuvant (CFA); day (D).

An inflammation-related increase in the production of reactive oxygen species was detected by bioluminescence imaging (Figure 3A,C). In the acute phase, we found a pronounced increase in the bioluminescent signal, which was substantially attenuated in the chronic phase. Nevertheless, the signal was still significantly elevated in the chronic phase (Figure 3B,D). Statistical analysis of the imaging data is described in Supplementary Tables S6–S9. These findings corroborate that the CFA-injected mice had sustained inflammatory arthritis.

### 3.3. Complete Blood Cell Counts Revealed Specific Cellular Changes of White Blood Cells

**Figure 4:**
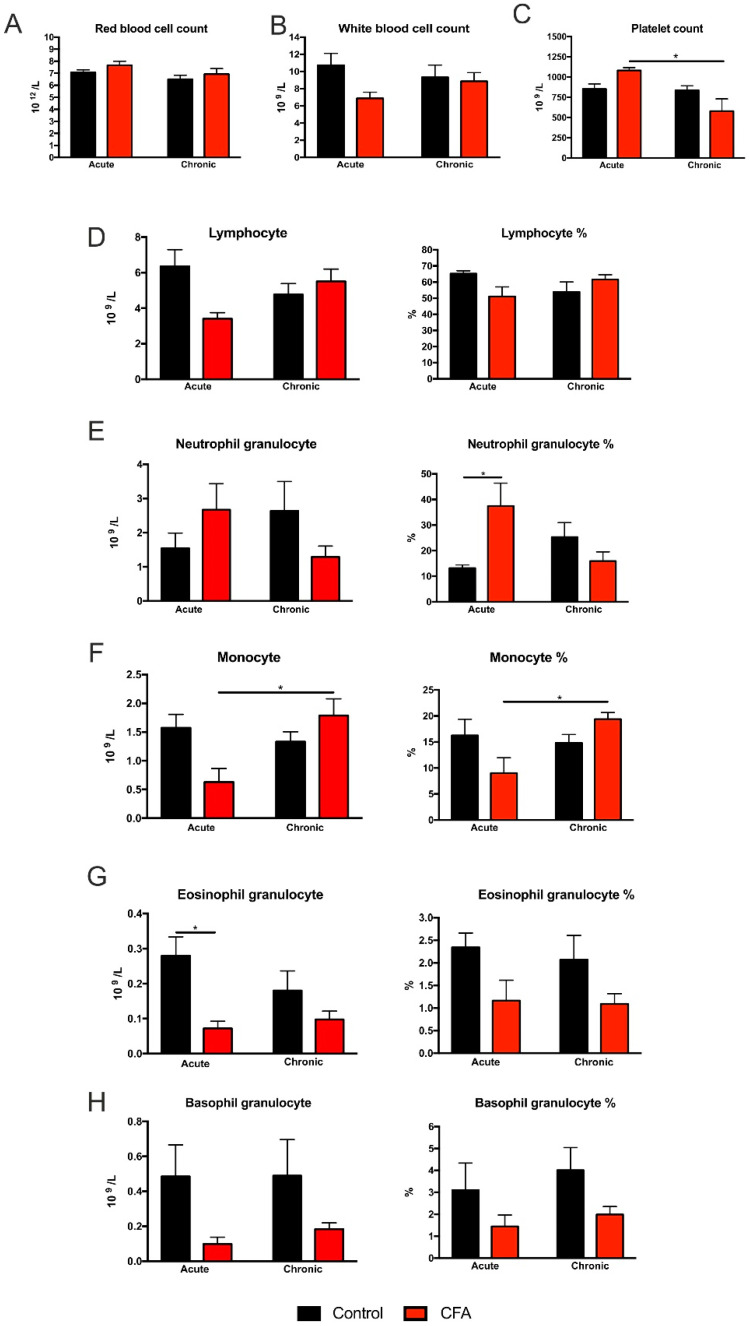
Inflammatory arthritis altered specific parameters of the complete blood cell count. Erythrocyte (**A**) and leukocyte cell numbers (**B**) were not influenced by the CFA treatment, but platelet numbers decreased in the Chronic CFA-treated animals compared to the acute phase (**C**). Lymphocyte cell numbers and percentage were not altered (**D**), but we found a significant increase in the neutrophil granulocyte ratio of the Acute CFA-treated animals (**E**). We also found a significant difference in monocyte cell numbers and the percentage between the Acute and Chronic CFA-treated animals (**F**). The total number of eosinophil granulocytes was reduced in the acute CFA-treated animals (**G**). Two-way ANOVA revealed a significant main effect of CFA-treatment on eosinophil and basophil granulocyte numbers and ratios (**G**,**H**). Statistical analysis: two-way ANOVA (time × CFA treatment) followed by Tukey’s multiple comparisons post hoc test. * *p* < 0.05. Abbreviations: complete Freund’s adjuvant (CFA); percentage (%).

Erythrocyte and leukocyte cell counts were not altered (Figure 4A,B), but platelet numbers decreased in the Chronic CFA-treated animals compared to the acute phase (*t* = 4.66, *p* < 0.05, Figure 4C). In the case of lymphocytes, a two-way ANOVA analysis revealed significant interaction between CFA-treatment and time (absolute numbers: *F* (1, 22) = 5.917, *p* < 0.05; percentage: *F* (1, 22) = 4.744, *p* < 0.05). Similarly, we detected a significant interaction between CFA-treatment and time for the neutrophil granulocyte ratios (percentage: *F* (1, 22) = 9.147, *p* < 0.01). We also found an increased neutrophil granulocyte ratio in the Acute CFA-treated animals (compared to the Acute Controls, *t* = 3.93, *p* < 0.05, Figure 4E).

In the case of monocytes, a two-way ANOVA revealed a significant time effect for monocyte percentage (*F* (1, 22) = 4.471, *p* < 0.05) as well as significant interaction between CFA-treatment and time (absolute numbers: *F* (1, 22) = 7.933, *p* < 0.01; percentage: *F* (1, 22) = 7.752, *p* = 0.01). CFA treatment increased monocyte cell numbers as well as monocyte percentage of the Chronic CFA-treated animals compared to the Acute CFA group (absolute numbers: *t* = 4.385, *p* < 0.05; percentage: *t* = 4.89, *p* = 0.01, Figure 4F).

CFA-treatment had a significant effect on eosinophil and basophil granulocytes. Two-way ANOVA revealed significant main treatment effects for eosinophils (absolute numbers: *F* (1, 22) = 9.569, *p* < 0.01; percentage: *F* (1, 22) = 6.195, *p* < 0.05) and also for basophils (absolute number: *F* (1, 22) = 4.783, *p* < 0.05); percentage: *F* (1, 22) = 6.883, *p* < 0.05) (Figure 4G,H). Furthermore, we found that the absolute number of eosinophil granulocytes significantly decreased in the Acute CFA-treated animals compared to the controls (*t* = 4.168, *p* < 0.05). For the detailed statistical analysis of the blood cell count data, please see Supplementary Tables S10–S22. 

In sum, the increased percentage of neutrophil granulocytes in the Acute CFA group implies a systemic acute inflammatory reaction.

### 3.4. Chronic CFA-Induced Arthritis Inhibits Adult Neurogenesis in the Hippocampal Dentate Gyrus

**Figure 5:**
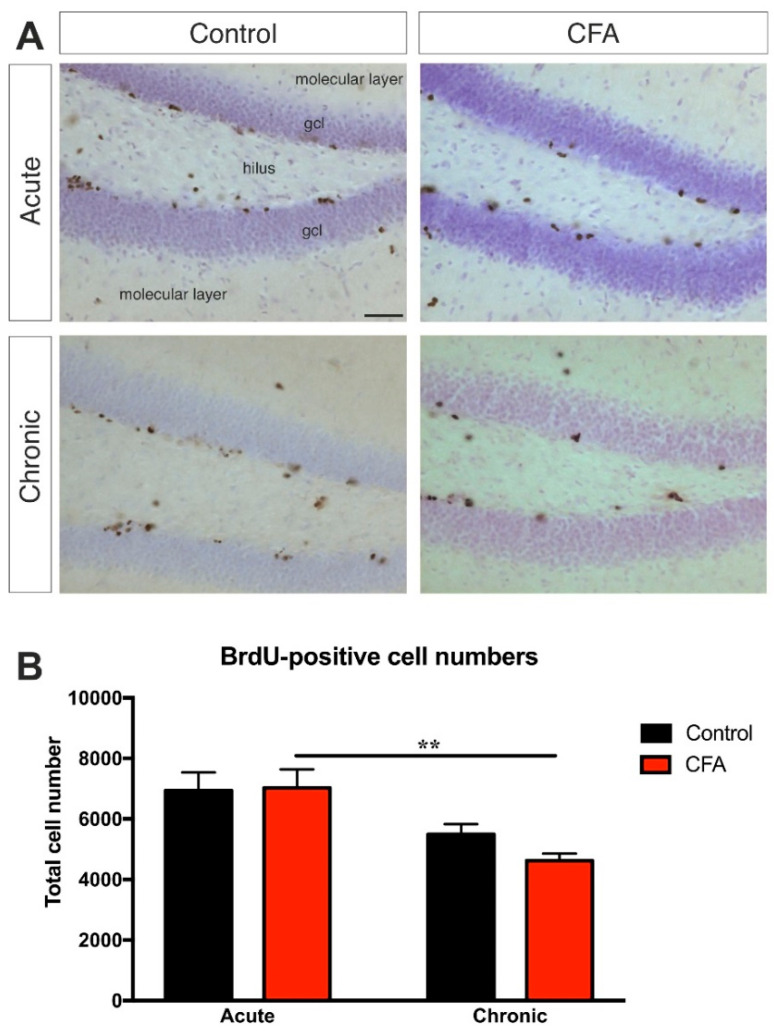
The effect of chronic arthritis on cell proliferation in the dentate gyrus. (**A**) Representative images demonstrating BrdU-immunopositive cells in the dentate gyrus of control and CFA-treated mice. Scale bar: 50 μm for all images. (**B**) Results of the systematic cell quantification data. Graphs represent the total number of BrdU-positive cells in both hemispheres combined. Animals in the chronic CFA treatment group had significantly reduced dentate cell proliferation compared to the acute CFA-treated group. Two-way ANOVA (time × CFA treatment) revealed a highly significant time effect but no effect of CFA-treatment, and the results of the post-hoc Tukey’s test is shown on the graph: ** *p* < 0.01. Abbreviations: 5-bromo-2′-deoxyuridine (BrdU); complete Freund’s adjuvant (CFA); granule cell layer (gcl).

Dentate cell proliferation was studied with quantitative anti-BrdU-immunohistochemistry. Acute inflammation had no influence on dentate cytogenesis, but, in the chronic inflammation group, we found a significantly lower number of newborn cells than in the acute phase (Figure 5). Two-way ANOVA (time × CFA treatment) revealed a highly significant time effect (*F*(1, 30) = 17.91, *p* < 0.001) but no effect of CFA-treatment. Post-hoc Tukey’s test revealed that Chronic CFA-treated mice had significantly reduced cell proliferation compared to the Acute CFA-treated group (*t* = 5.28, *p* < 0.01). This reduction of BrdU+ cell numbers in the Chronic CFA group was most likely the combined effect of age and CFA treatment, since there was no significant difference between the Acute Control and Chronic Control groups, which suggests that the difference between the Acute CFA and Chronic CFA groups is not simply an ageing effect. However, the number of BrdU+ cells were not different in the Chronic Control and Chronic CFA-treated groups. For the detailed statistical analysis, see Supplementary Table S23.

**Figure 6:**
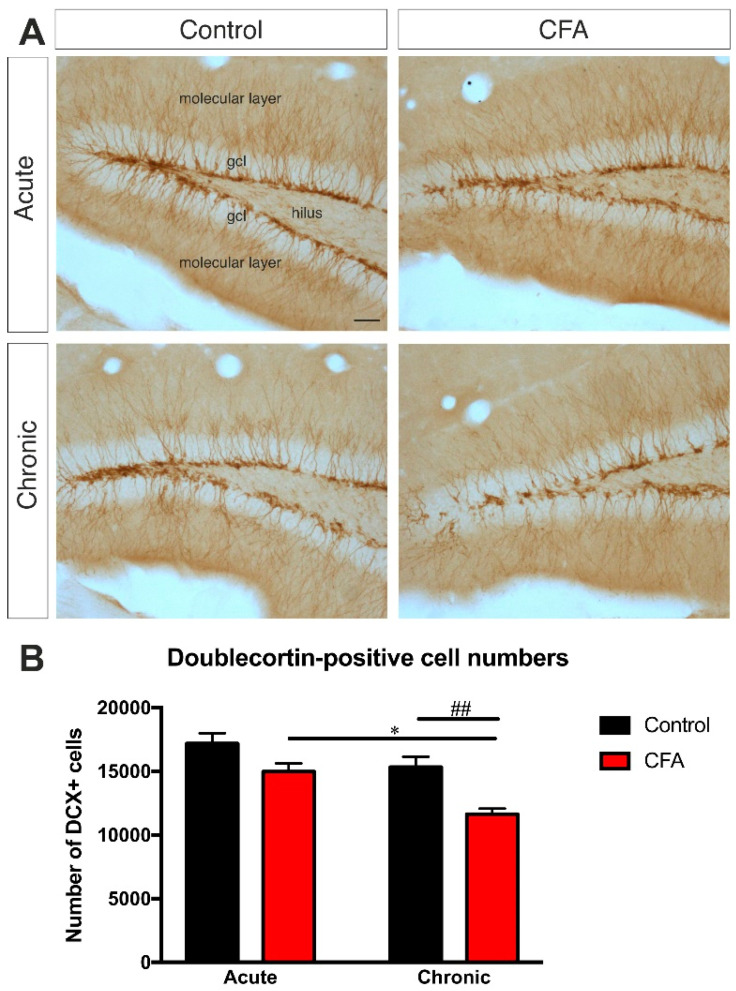
Chronic inflammatory arthritis inhibits adult neurogenesis in the dentate gyrus. (**A**) Representative images demonstrating doublecortin-immunopositive immature neurons in the dentate gyrus of control and CFA-treated mice. Scale bar: 50 μm for all images. (**B**) Results of the systematic cell quantification data. Graphs represent the total number of doublecortin-positive cells in the dentate gyrus of both hemispheres combined. Animals in the CFA-induced chronic arthritis group had a significantly reduced number of DCX-positive cell numbers. Statistics: two-way ANOVA (time × CFA treatment) followed by Tukey’s multiple comparisons post hoc test. * *p* < 0.05 versus the Acute CFA-treated group; ## *p* < 0.01 versus the Chronic Control group. Abbreviations: complete Freund’s adjuvant (CFA); doublecortin (DCX); granule cell layer (gcl).

The number of newborn immature neurons was investigated with quantitative anti-doublecortin-immunohistochemistry. DCX is a widely used immuno-marker for immature neurons [52]. It is expressed just a few hours after neurogenesis, and the peak of its expression level is reached between Days 4–7 after neuronal birth [51]. Chronic arthritis reduced the number of doublecortin-positive cells in the hippocampus of the Chronic CFA animals (Figure 6). Acute inflammation had only a tendency to reduce dentate neurogenesis, but, in the chronic inflammation group, we found a significantly lower number of newborn granule cells. Two-way ANOVA (time × CFA treatment) revealed a significant CFA treatment effect (*F*(1, 30) = 17.89, *p* < 0.001) as well as a significant time effect (*F*(1, 30) = 13.82, *p* < 0.001). Post-hoc comparison with Tukey’s test revealed that Chronic CFA-treated mice had significantly reduced doublecortin-positive cells compared to the Chronic Control (*t* = 5.47, *p* < 0.01) and Acute CFA-treated groups (*t* = 4.79, *p* = 0.01). This reduction of doublecortin+ cell numbers of the Chronic CFA group demonstrates the inhibitory effect of chronic arthritis on adult hippocampal neurogenesis. For the detailed statistical analysis of the doublecortin+ cell number data, see Supplementary Table S24.

### 3.5. Peripheral Inflammation Activated Macrophages and Microglia in the Dentate Gyrus

**Figure 7:**
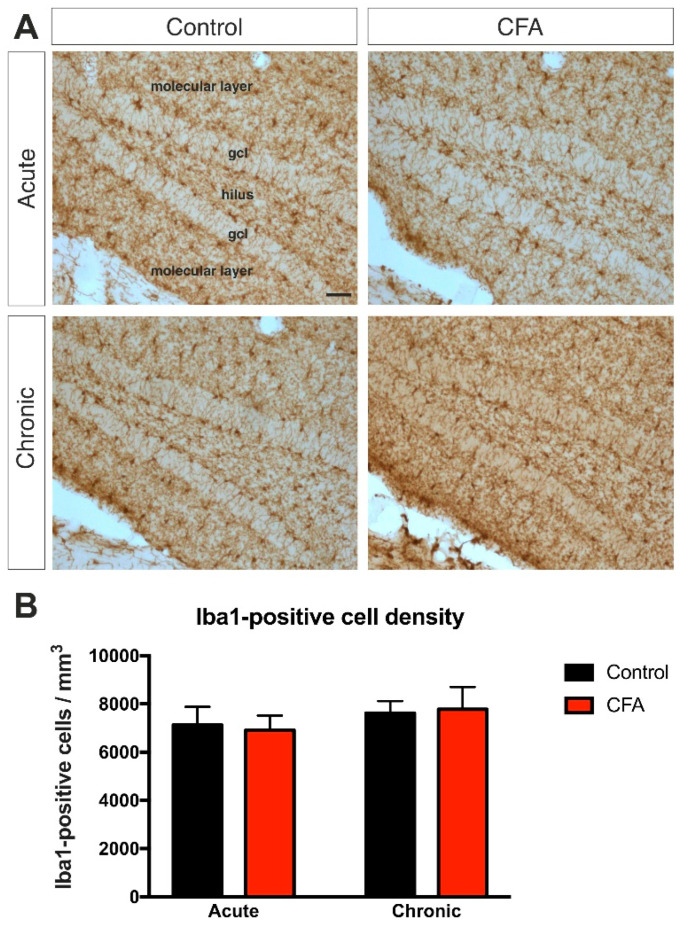
Inflammatory arthritis had no effect on the number of Iba1-immunopositive microglia in the dentate gyrus. (**A**) Representative images demonstrating Iba1-immunopositive microglia in the dentate gyrus of control and CFA-treated mice. Scale bar: 50 μm for all images. (**B**) Results of the systematic cell quantification data. Graphs represent the density of Iba1-positive cells (Iba1+ cell number/mm^3^) Statistics: two-way ANOVA (time × CFA treatment) followed by Tukey’s multiple comparisons post hoc test. Abbreviations: complete Freund’s adjuvant (CFA); granule cell layer (gcl); ionized calcium binding adaptor molecule-1 (Iba1).

Since activated microglia are prime cellular candidates for regulating adult hippocampal neurogenesis [15,22,24,59,60], we therefore also quantified the number of Iba1-positive microglia in the dentate gyrus. We did not find any treatment effect on Iba1+ microglial cell numbers (Figure 7, the detailed statistical analysis is shown in Supplementary Table S25).

**Figure 8:**
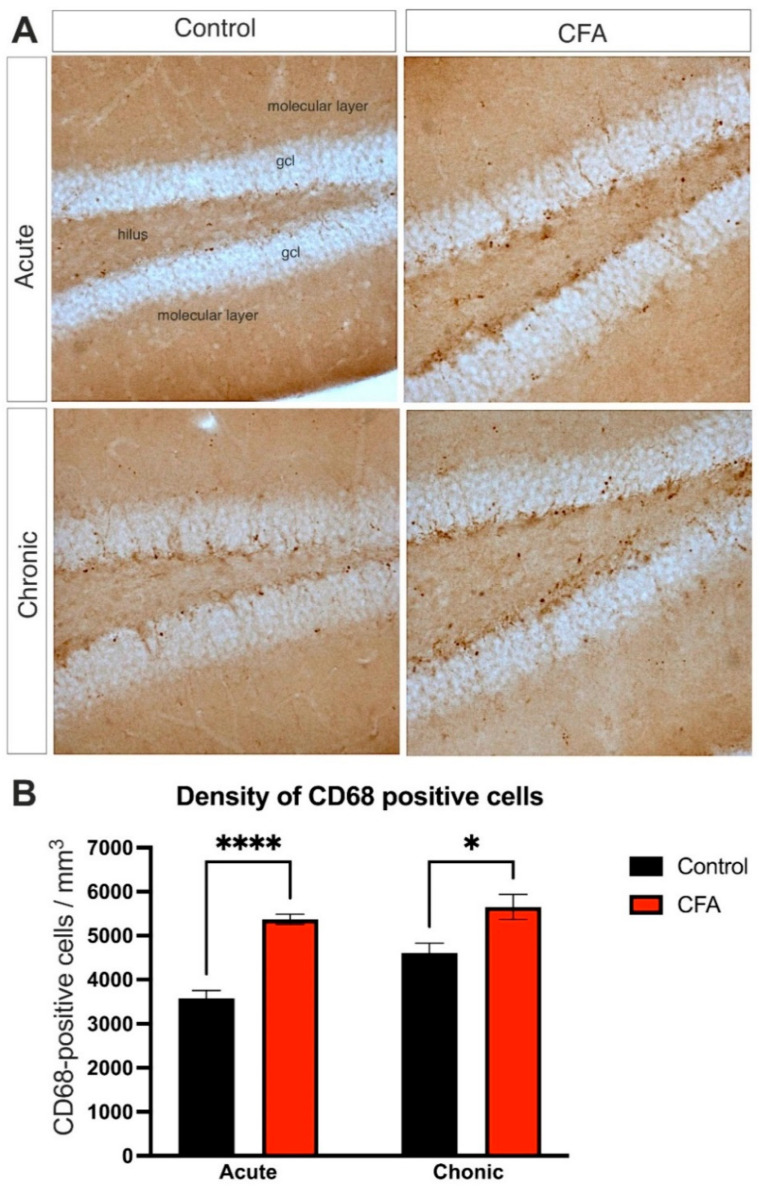
Inflammatory arthritis increased the number of CD68-immunopositive macrophages/activated microglia in the dentate gyrus. (**A**): Representative images demonstrating CD68+ microglia and macrophages in the dentate gyrus of control and CFA-treated mice. Magnification 20× for all images. (**B**): Results of the cell quantification data. Graphs represent the density of CD68-positive cells (CD68+ cell number/mm^3^). Statistics: two-way ANOVA (time × CFA treatment) followed by Tukey’s multiple comparisons post hoc test. Abbreviations: complete Freund’s adjuvant (CFA); granule cell layer (gcl). * *p* < 0.05; **** *p* < 0.0001 versus control values at the same time point.

We also carried out an immunostaining to visualize CD68-positive cells in the dentate gyrus. CD68 is a glycoprotein that is mainly located in the endosomal/lysosomal compartment and strongly expressed in macrophages and other mononuclear phagocytes [61]. Therefore, it is typically used as a cytochemical marker to visualize monocyte/macrophages in the histochemical analysis of inflamed tissues. In contrast to the Iba1 data, we found a significantly increased density of the CD68-positive cells in the dentate gyrus (Figure 8). Two-way ANOVA (time × CFA treatment) revealed a significant time effect (*F*(1, 23) = 8.397, *p* < 0.01), as well as a highly significant CFA-treatment effect (*F*(1, 23) = 39.68, *p* < 0.0001), but no interaction. Post-hoc Tukey’s test revealed that, in the Acute CFA-treated mice, CD68+ cell density was significantly increased compared to the Acute Control group (*t* = 7.83, *p* < 0.0001). Similarly, CD68+ cell numbers were significantly increased in the Chronic CFA group compared to the Chronic Control group (*t* = 4.72, *p* < 0.05) (Figure 8B). For the detailed statistical analysis, see Supplementary Table S26.

### 3.6. CFA-Treatment Increased Inflammatory Cytokine Levels in the Inflamed Hind Paws, but Had Little or No Effect in the Peripheral Blood and Hippocampus

**Figure 9:**
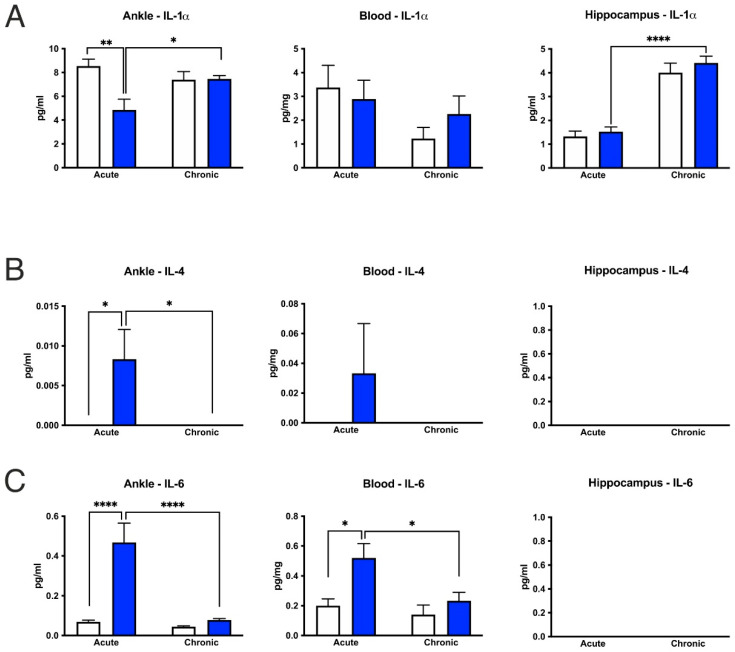

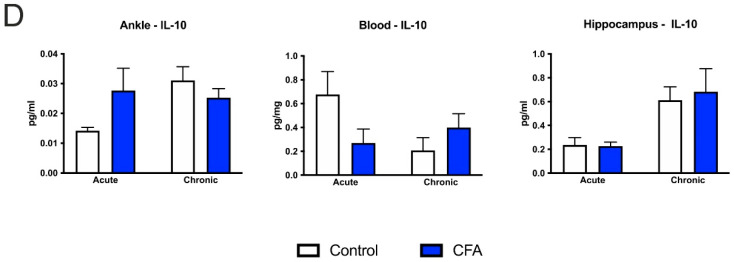
Cytokine/chemokine concentrations in the CFA-treated hind paws, peripheral blood and hippocampus. (**A**–**C**) In the hind paws, acute inflammation increased IL-4 and IL-6 concentrations and decreased IL-1α levels. (**C**) In the peripheral blood, only IL-6 levels increased in response to acute inflammation. In the hippocampus, CFA treatment did not alter any cytokine levels compared to the corresponding control groups. (**D**) IL-10 concentrations were not altered in any tissue by the CFA-treatment. On some graphs, there are no bars, which means that those cytokine concentrations were below the detection levels. Statistics: two-way ANOVA (CFA treatment × time) followed by Tukey’s multiple comparisons post hoc test. * *p* < 0.05; ** *p* < 0.01; **** *p* < 0.0001. Abbreviations: complete Freund’s adjuvant (CFA); interleukin-1-alpha (IL-1α); interleukin-4 (IL-4); interleukin-6 (IL-6); interleukin-10 (IL-10).

**Figure 10:**
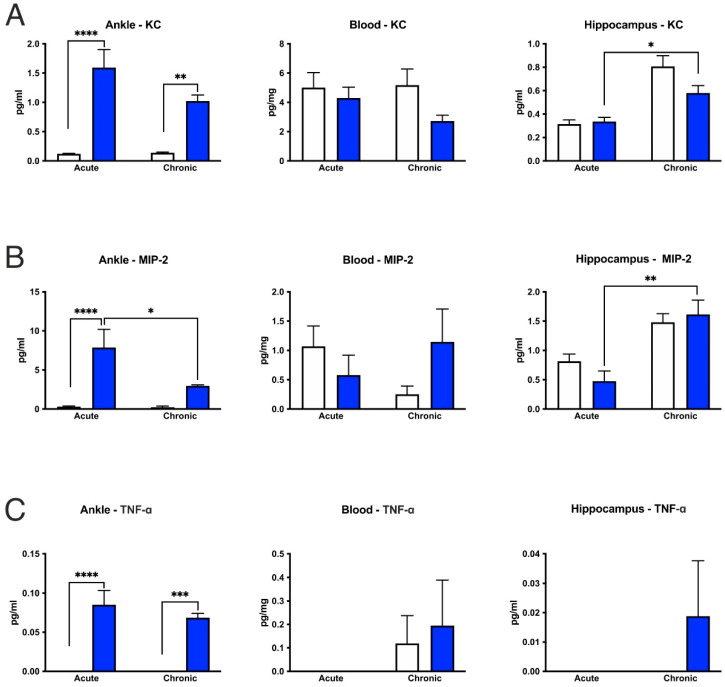

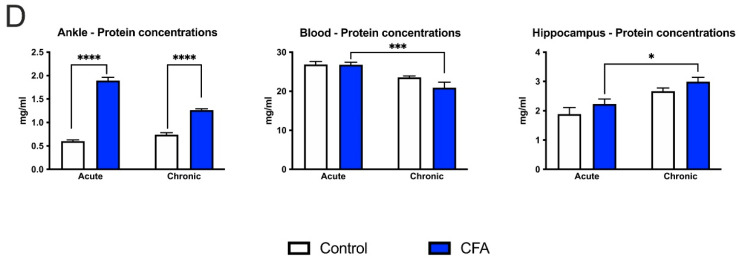
Cytokine/chemokine and total protein concentrations in the CFA-treated hind paws, peripheral blood and hippocampus. (**A**–**C**) In the hind paws, acute inflammation increased cytokine concentrations of KC, MIP-2 and TNF-α, whereas chronic arthritis increased KC and TNF-α levels. In the peripheral blood, we found no change in these cytokine levels. In the hippocampus, CFA treatment did not alter any cytokine levels compared to the corresponding control groups. (**D**) Both acute and chronic inflammation increased total protein concentrations in the joints, and a similar trend was present in the hippocampus. On some graphs, there are no bars, which means that those cytokine concentrations were below the detection levels. Statistics: two-way ANOVA (CFA treatment × time) followed by Tukey’s multiple comparisons post hoc test. * *p* < 0.05; ** *p* < 0.01; *** *p* < 0.001; **** *p* < 0.0001. Abbreviations: complete Freund’s adjuvant (CFA); keratinocyte-derived chemokine (KC); macrophage inflammatory protein-2 (MIP-2); tumor necrosis factor alpha (TNF-α).

In the hind limbs, acute CFA treatment increased the cytokine concentrations of IL-4, IL-6, KC, MIP-2 and TNF-α, whereas the IL-1α levels decreased. In the chronic inflammation phase, only the TNF-α and KC levels remained elevated (Figure 9 and Figure 10). Similarly, both acute and chronic inflammation induced a pronounced increase in the total protein concentrations in the hind paws (Figure 10D). In the peripheral blood samples, most of the measured cytokine levels were not altered in the CFA-treated animals, with the exception of an increase in the IL-6 concentration in the acute CFA-treated group (Figure 9C). In the hippocampus, cytokine levels were not altered by the CFA treatment (Figure 9 and Figure 10). Although we found significant differences in the cytokine levels of IL-1α, KC, MIP-2 and protein concentrations between the acute and chronic CFA-treated animals, when comparing the cytokine levels in the hippocampus between the CFA-treated mice and their corresponding controls, we could not detect any statistically significant difference (Figure 9 and Figure 10). For the detailed statistical analysis, see Supplementary Tables S27–S48.

## 4. Discussion

Here, we report that chronic CFA-induced arthritis can inhibit neurogenesis in the adult hippocampus. Adult neurogenesis is a complex, multistep process, which involves cell proliferation, cell differentiation and maturation, as well as the integration of newborn neurons into the pre-existing circuit [62]. In the present experiment, cell proliferation (i.e., the number of BrdU+ cells) was not influenced by the peripheral inflammation, and the survival of newborn neurons (i.e., the number of DCX cells) was reduced in the CFA-treated animals. In other words, the CFA-induced changes in neurogenesis occurred during the early maturation phase of the newborn neurons, rather than during the proliferation phase.

In our CFA-induced experimental arthritis model, we found clear molecular, cellular and behavioral evidence of the acute and chronic peripheral inflammation in the hind paws. In the acute inflammatory phase, the blood cell count data suggested a more generalized, systemic inflammatory reaction, but this was not reflected in the cytokine/chemokine levels of the peripheral blood, except for IL-6, which increased. In the chronic phase, we found no evidence of a systemic inflammatory reaction in blood cell numbers or serum cytokine/chemokine concentrations. In the hippocampus, we found no change in the cytokine/chemokine levels, and the Iba1-positive microglia numbers also remained unaltered both in the acute and chronic inflammatory phase. However, we found a significantly increased density of the CD68-positive macrophages/activated microglia in the dentate gyrus of the CFA-treated mice, both in the acute and chronic inflammatory phases. Overall, these data suggest that acute peripheral inflammation initiates a cascade of molecular and cellular changes that eventually leads to reduced adult neurogenesis in the dentate gyrus since essentially all of the inflammatory reactions that we could detect in the blood were present only in the early phase of inflammation.

Previous studies reported controversial findings regarding the effect of peripheral inflammation on adult hippocampal neurogenesis. Data from experimental colitis models documented either inhibition of adult neurogenesis [30,31] or normal levels with deficits in the migration and integration of newborn neurons in the functional circuitry of the dentate gyrus [32], whereas experimental arthritis models reported either a transient increase in hippocampal precursor cell proliferation and neurogenesis [33] or no effect [34].

It is increasingly acknowledged that chronic systemic inflammations can contribute to the development and worsening of mental disorders, including depression and neurodegeneration [63,64,65,66]. One explanatory concept is that the systemic pro-inflammatory cytokines/chemokines can cause a breach in the blood brain-barrier and thereby enable the entry of immune cells and pro-inflammatory cytokines/chemokines into the brain. These peripheral-derived factors, together with the intrinsically generated cytokines/chemokines, can activate glial cells, which in turn trigger additional release of inflammatory and neurotoxic molecules that contribute to the development of neuroinflammation and neurodegeneration [66]. Indeed, a recent study reported that animals with chronic colitis had a parallel increase in the cytokine levels of TNF-alpha, IL-1-beta, IL-6 and IL-10 in their blood and hippocampus [32].

In our CFA-induced arthritis model, we found clear evidence of acute and chronic peripheral inflammation. We documented local inflammation with bioluminescence imaging, and arthritis-related hypersensitivity to painful stimuli was revealed by the behavioral tests. Results of the complete blood cell counts also indicated inflammation at the periphery. Our hypothesis was that inflammatory arthritis should induce a parallel increase in cytokine levels in the joints, peripheral blood and the hippocampus, as well, but we could not confirm that. However, we did find a pronounced elevation of protein concentrations in the inflamed joints, and a similar trend was present in the hippocampus of the CFA-treated animals. Most likely, this increase in total protein concentrations was due to the elevated production of inflammatory mediators.

We focused on specific cytokines (IL-1α, IL-4, IL-6, IL10, TNF-α, KC, MIP-2) since previous studies documented that these inflammatory mediators can influence neurogenesis in adult dentate gyrus. The IL-1 family is a potent group of pro-inflammatory cytokines, and elevated brain IL-1 levels can suppress adult neurogenesis [16,67,68,69,70]. IL-4 is an anti-inflammatory cytokine that can activate microglia and regulates the M1/M2 polarization of microglial cells [71,72]. Via these cellular effects, it can also eventually influence the production of neurons [73]. IL-6 is a pro-inflammatory cytokine produced at the site of inflammation and plays a central role in the acute phase response. It is a major cytokine in the central nervous system [74] and a potent suppressor of neurogenesis [14,15]. IL-10 plays a complex role in the regulation of the immune response, but it is typically regarded as an anti-inflammatory cytokine. It regulates neurogenesis in a complex manner. It maintains neural progenitors in an undifferentiated state by keeping the progenitors in an active cycle, reducing neuronal differentiation, and, ultimately, it seems to inhibit the endogenous production of newborn neurons [75]. TNF-α is another potent pro-inflammatory cytokine, typically increased in the acute phase of inflammation, and it plays a central role in initiating the cascade of other cytokines. Numerous studies investigated the effect of TNF-α on neurogenesis, most data document inhibition [76,77,78], while some report on a pro-neurogenic effect [79,80]. Furthermore, peripherally induced inflammation promotes the transient activation of adult neural stem cells, and TNF-α seems to mediate this effect [81]. MIP-2 and KC are members of the platelet-factor 4 cytokine superfamily, and they both play a key role in the local inflammatory reaction as well as in the chemotaxis and infiltration of polymorphonuclear leukocytes [82,83]. To the best of our knowledge, their role in adult neurogenesis has not yet been investigated.

A major limitation of our study is that it was a cross-sectional design with only two time points. Therefore, we cannot rule out the possibility that a more pronounced neuroinflammation response was present at earlier time points. Other molecular and/or cellular inflammatory factors—which were not studied here—may also play a significant role in the inhibitory effect of chronic arthritis on adult neurogenesis. Another possibility is that the reduced rate of adult neurogenesis was modulated by nociceptive input from the hind limb to the contralateral hemisphere. In this case, one would expect a unilateral change, i.e., decreased neurogenesis in the contralateral hippocampus. Since our original hypothesis was that circulating cytokines/chemokines should mediate the inhibitory effect of peripheral inflammation, we therefore did not focus on potential hemispheric asymmetries in adult hippocampal neurogenesis. Circulating signal mediators are unlikely to induce a unilateral effect. An important argument against the regulatory effect of direct sensory pain signal transduction is that the earlier studies, which used unilateral pain models, could not find any hemispheric differences in the rate of adult neurogenesis [36,37,84,85]. This again suggests that circulating signals mediate the inhibitory effect.

The presence and functional significance of adult neurogenesis in the human hippocampus are controversial and extensively debated issues [2,86,87,88,89]. Disturbed production of newborn neurons has been implicated in a large variety of mental and neurological disorders [13,90,91]. Microglial cells have been pointed out as potent local regulators of neurogenesis [22,92,93], but their exact regulatory role depends on numerous factors. Acutely activated microglia have different effects from chronically activated cells, and they can be further divided into different subpopulations based on their functional phenotype. Therefore, different microglial cells may even exert opposite effects (supportive or detrimental) on adult neurogenesis, and they act on the different stages of neurogenesis, i.e., cell formation, maturation and functional integration of the newborn neurons [22,94]. Notably, a very recent systematic review concluded that the microglial changes induced by environmental stress seem to regulate neurogenesis and, in turn, may be responsible for the development of depressive-like behaviors, but the available data are not entirely conclusive, and other factors should not be dismissed [60]. Previous studies found an increased number and hypertrophy of activated microglia, as well as increased expression of the Iba1-protein in the hippocampi of mice with DSS-induced colitis [30,31]. Intraperitoneal administration of LPS can also activate hippocampal microglia [29]. Here, we found an increased density of the CD68+ macrophages/activated microglia in the hippocampus, but we did not see any change in the Iba1+ microglial density after the CFA treatment. CD68 is a glycoprotein that is mainly located in the endosomal/lysosomal compartment and strongly expressed in macrophages and other mononuclear phagocytes. Therefore, it is typically used as a cytochemical marker for visualization of monocyte/macrophages in the histochemical analysis of inflamed tissues [61]. However, in some studies, it is used as a marker for activated microglia [95]. Results of comparative studies suggest that the different microglial markers have different potential for neuropathological analysis, i.e., CD68-positivity reflects immune activation and response to tissue damage, whereas Iba1 is a marker that is more suited for structural studies in the absence of pathology [96]. Yet another study documented that the CD68+ small, round cells in the damaged brain tissue express specific cellular markers, indicating that these cells are in fact not activated microglia but neutrophils [97]. Interestingly, post-mortem clinical studies also report on increased number of CD68+ cells in the hippocampi and neocortices of patients who died in septic shock [98,99].

## 5. Conclusions 

Here, we report that chronic, but not acute, arthritis can inhibit adult hippocampal neurogenesis. Since almost all the inflammatory reactions in the circulating blood were found in the early phase of the inflammation, our present data therefore suggest that the key regulatory mechanisms are in fact triggered during the early inflammatory phase when a cascade of molecular and cellular events are initiated, which will eventually lead to reduced adult hippocampal neurogenesis. However, this negative impact on the hippocampus is detectable only several weeks later during the chronic inflammatory phase.

## Data Availability

The data presented in this study are available on request from the corresponding author. The data are not publicly available due to privacy concerns.

## Supplementary Materials

The following are available online at https://www.mdpi.com/article/10.3390/cells11050791/s1, Supplementary Table S1: Statistical analysis of data generated with the dynamic plantar aesthesiometry test. Table S2: Statistical analysis of data generated using the hot plate test. Table S3: Statistical analysis of data generated by measuring hind paw volumes of the animals. Table S4: Statistical analysis of data obtained by measuring spontaneous bodyweight distribution of the acute CFA treated animals. Table S5: Statistical analysis of data obtained by measuring spontaneous bodyweight distribution of the chronic CFA treated animals. Table S6: Statistical analysis of the in vivo bioluminescent data detecting macrophage NADPH oxidase activity in the animals with acute CFA treatment. Table S7: Statistical analysis of the in vivo bioluminescent data detecting macrophage NADPH oxidase activity in the animals with chronic CFA treatment. Table S8: Statistical analysis of the in vivo bioluminescent data detecting the myeloperoxidase enzyme activity of neutrophil granulocytes in the animals of the acute inflammatory phase. Table S9: Statistical analysis of the in vivo bioluminescent data detecting the myeloperoxidase enzyme activity of neutrophil granulocytes in the animals of the chronic inflammatory phase. Table S10: Statistical analysis of the red blood cell count data. Table S11: Statistical analysis of the white blood cell count data. Table S12: Statistical analysis of the platelet count data. Table S13: Statistical analysis of the lymphocyte absolute number data. Table S14: Statistical analysis of the lymphocyte percentage data. Table S15: Statistical analysis of the neutrophil granulocyte absolute number data. Table S16: Statistical analysis of the neutrophil granulocyte percentage data. Table S17: Statistical analysis of the monocyte absolute number data. Table S18: Statistical analysis of the monocyte percentage data. Table S19: Statistical analysis of the eosinophil granulocyte absolute number data. Table S20: Statistical analysis of the eosinophil granulocyte percentage data. Table S21: Statistical analysis of the basophil granulocyte absolute number data. Table S22: Statistical analysis of the basophil granulocyte percentage data. Table S23: Statistical analysis of the BrdU-positive cell number data. Table S24: Statistical analysis of the doublecortin-positive cell number data. Table S25: Statistical analysis of the Iba1-positive cell number data. Table S26: Statistical analysis of the CD68-positive microglia / macrophages cell number data. Table S27: Statistical analysis of the IL-1α cytokine concentration data in the hind paws. Table S28: Statistical analysis of the IL-1α cytokine concentration data in the peripheral blood. Table S29: Statistical analysis of the IL-1α cytokine concentration data in the hippocampus. Table S30: Statistical analysis of the IL-4 cytokine concentration data in the hind paw. Table S31: Statistical analysis of the IL-4 cytokine concentration data in the peripheral blood. Table S32: Statistical analysis of the IL-6 cytokine concentration data in the hind paws. Table S33: Statistical analysis of the IL-6 cytokine concentration data in the peripheral blood. Table S34: Statistical analysis of the IL-10 cytokine concentration data in the hind paws. Table S35: Statistical analysis of the IL-10 cytokine concentration data in the peripheral blood. Table S36: Statistical analysis of the IL-10 cytokine concentration data in the hippocampus. Table S37: Statistical analysis of the KC concentration data in the hind paws. Table S38: Statistical analysis of the KC concentration data in the peripheral blood. Table S39: Statistical analysis of the KC concentration data in the hippocampus. Table S40: Statistical analysis of the MIP-2 concentration data in the hind paws. Table S41: Statistical analysis of the MIP-2 concentration data in the peripheral blood. Table S42: Statistical analysis of the MIP-2 concentration data in the hippocampus. Table S43: Statistical analysis of the TNF-α concentration data in the hind paws. Table S44: Statistical analysis of the TNF-α concentration data in the peripheral blood. Table S45: Statistical analysis of the TNF-α concentration data in the hippocampus. Table S46: Statistical analysis of the protein concentration data in the hind paws. Table S47: Statistical analysis of the protein concentration data in the peripheral blood. Table S48: Statistical analysis of the protein concentration data in the hippocampus.

## Abbreviations

ADWB: advanced dynamic weight bearing; BL: baseline; BrdU: 5-bromo-2′-deoxyuridine; CFA: complete Freund’s adjuvant; DCX: doublecortin; DPA: dynamic plantar aesthesiometry; DSS: dextran sulfate sodium; gcl: granule cell layer; Iba1: ionized calcium binding adaptor molecule-1; IL-1α: interleukin-1-alpha; IL-4: interleukin-4; IL-6: interleukin-6; IL-10: interleukin-10; KC: keratinocyte-derived chemokine; LPS: lipopolysaccharide; MIP-2: macrophage inflammatory protein-2; MPO: myeloperoxidase; Phox: macrophage NADPH oxidase; TNF-α: tumor necrosis factor alpha.
