# Supplementary material for: Experimental Arthritis Inhibits Adult Hippocampal Neurogenesis in Mice
Source: Cells. 2022 Feb 24;11(5):791. doi: 10.3390/cells11050791 (PMC8909078; doi:10.3390/cells11050791)
Supplement: Supplementary file 1 [file cells-11-00791-s001.zip › CELLS 1604080 Rusznak_Chronic inflammation and AN Supplementary materials 2022.02.25..pdf]

<u>**Supplementary information for**</u>

Chronic arthritis inhibits adult hippocampal neurogenesis in mice.

Kitti Rusznák, Ádám István Horváth, Kinga Pohli-Tóth, Anett Futácsi, Ágnes Kemény, Gabriella Kiss, Zsuzsanna Helyes and Boldizsár Czéh *

*** Correspondence:** Boldizsár Czéh, E-mail: czeh.boldizsar@pte.hu

Supplementary Table S1.

Statistical analysis of data generated with the dynamic plantar aesthesiometry test.

Dynamic plantar aesthesiometer					
Acute Control *versus* Acute CFA treatment groups			Chronic Control *versus* Chronic CFA treatment groups		
Two-way repeated measures ANOVA					
**Main treatment effects**	***F* (DFn, DFd) value**	***P* value**	**Main treatment effects**	***F* (DFn, DFd) value**	***P* value**
**Time effect**	*F* (2, 20) = 17,57	<0,0001	**Time effect**	*F* (6, 84) = 8,096	P<0,0001
**CFA treatment effect**	*F* (1, 10) = 262,4	<0,0001	**CFA treatment effect**	*F* (1, 14) = 397,7	P<0,0001
**Interaction: Time × CFA treatment**	*F* (2, 20) = 27,09	<0,0001	**Interaction: Time × CFA treatment**	*F* (6, 84) = 12,33	P<0,0001
**Sidak's multiple comparisons test**					
**Control *versus* CFA**	***t* value**	***P* value**	**Control *versus* CFA**	***t* value**	***P* value**
**Baseline (BL)**	0,3127	0,9858	**Baseline (BL)**	1,536	0,6177
**Day 3**	9,172	<0,0001	**Day 3**	12,41	<0,0001
**Day 7**	8,146	<0,0001	**Day 7**	8,134	<0,0001
			**Day 11**	10,91	<0,0001
			**Day 15**	8,843	<0,0001
			**Day 18**	9,256	<0,0001
			**Day 21**	10,56	<0,0001

This behavioral data is shown on Figure 2A-B.

Supplementary Table S2.

Statistical analysis of data generated using the hot plate test.

Hot plate test					
**Acute Control *versus* Acute CFA treatment groups**			**Chronic Control *versus* Chronic CFA treatment groups**		
**Two-way repeated measures ANOVA**					
**Main treatment effects**	***F* (DFn, DFd) value**	***P* value**	**Main treatment effects**	***F* (DFn, DFd) value**	***P* value**
**Time effect**	*F* (3, 30) = 5,368	0,0044	**Time effect**	*F* (5, 140) = 11,19	<0,0001
**CFA treatment effect**	*F* (1, 10) = 4,46	0,0608	**CFA treatment effect**	*F* (1, 28) = 121,9	<0,0001
**Interaction: Time × CFA treatment**	*F* (3, 30) = 2,223	0,1059	**Interaction: Time × CFA treatment**	*F* (5, 140) = 15,17	<0,0001
**Sidak's multiple comparisons test**					
**Control *versus* CFA**	***t* value**	***P* value**	**Control *versus* CFA**	***t* value**	***P* value**
**Baseline (BL)**	0,03072	>0,9999	**Baseline (BL)**	0,7312	0,9771
**Day 1**	2,388	0,0905	**Day 1**	2,569	0,0718
**Day 4**	3,547	0,0052	**Day 4**	4,816	<0,0001
**Day 7**	2,041	0,1859	**Day 7**	3,532	0,0044
			**Day 17**	8,981	<0,0001
			**Day 21**	8,816	<0,0001

The behavioral data is shown on Figure 2C-D.

Supplementary Table S3.

Statistical analysis of data generated by measuring hind paw volumes of the animals.

Hind Paw volume					
**Acute Control *versus* Acute CFA treatment groups**			**Chronic Control *versus* Chronic CFA treatment groups**		
**Two-way repeated measures ANOVA**					
**Main treatment effects**	***F* (DFn, DFd) value**	***P* value**	**Main treatment effects**	***F* (DFn, DFd) value**	***P* value**
**Time effect**	*F* (4, 40) = 56,71	<0,0001	**Time effect**	*F* (9, 126) = 68,23	<0,0001
**CFA treatment effect**	*F* (1, 10) = 310,8	<0,0001	**CFA treatment effect**	*F* (1, 14) = 490,8	<0,0001
**Interaction: Time × CFA treatment**	*F* (4, 40) = 57,23	<0,0001	**Interaction: Time × CFA treatment**	*F* (9, 126) = 68,84	<0,0001
**Sidak's multiple comparisons test**					
**Control *versus* CFA**	***t* value**	***P* value**	**Control *versus* CFA**	***t* value**	***P* value**
**Baseline (BL)**	0,369	0,9981	**Baseline (BL)**	0,301	>0,9999
**Day 1**	10,7	<0,0001	**Day 1**	11,24	<0,0001
**Day 3**	15,13	<0,0001	**Day 3**	14,85	<0,0001
**Day 5**	16,24	<0,0001	**Day 5**	21,07	<0,0001
**Day 7**	18,82	<0,0001	**Day 7**	23,88	<0,0001
			**Day 9**	23,48	<0,0001
			**Day 11**	24,48	<0,0001
			**Day 15**	27,09	<0,0001
			**Day 18**	25,08	<0,0001
			**Day 21**	22,68	<0,0001

Data is shown on Figure 2E-F.

Supplementary Table S4.

Statistical analysis of data obtained by measuring spontaneous bodyweight distribution of the acute CFA treated animals.

Advanced Dynamic Weight Bearing test of the CFA-treated animals (Acute CFA group)		
**Two-way ANOVA**		
**Main effects**	***F* (DFn, DFd) value**	***P* value**
**Time effect**	F (1, 36) = 0,000	>0,9999
**CFA treatment effect**	F (1, 36) = 57,68	<0,0001
**Interaction: Time × CFA treatment**	F (1, 36) = 59,33	<0,0001
**Tukey's multiple comparisons test**		
**Right hindlimb - Left hindlimb**	***t* value**	***P* value**
BL	0,2517	0,9608
D7	10,82	<0,0001

Data is shown on Figure 2G.

Supplementary Table S5.

Statistical analysis of data obtained by measuring spontaneous bodyweight distribution of the chronic CFA treated animals.

Advanced Dynamic Weight Bearing test of the CFA-treated animals (Chronic CFA group)		
**Two-way ANOVA**		
**Main effects**	***F* (DFn, DFd) value**	***P* value**
**Time effect**	F (1, 28) = 0,000	>0,9999
**CFA treatment effect**	F (1, 28) = 1,364	0,2526
**Interaction: Time × CFA treatment**	F (1, 28) = 7,575	0,0103
**Tukey's multiple comparisons test**		
**Right hindlimb - Left hindlimb**	***t* value**	***P* value**
BL	1,231	0,3972
D21	2,815	0,0134

Data is shown on Figure 2H.

Supplementary Table S6.

Statistical analysis of the *in vivo* bioluminescent data detecting macrophage NADPH oxidase activity in the animals with acute CFA treatment.

**Macrophage NADPH oxidase activity**		
**Two-way ANOVA**		
**Main effects**	***F* (DFn, DFd) value**	***P* value**
**Contralateral *versus* ipsilateral**	*F* (1, 86) = 29,04	<0,0001
**CFA treatment effect**	*F* (1, 86) = 27,5	<0,0001
**Interaction**	*F* (1, 86) = 28,89	<0,0001
**Tukey's multiple comparisons test**		
**Contralateral *versus* ipsilateral**	***t* value**	***P* value**
Control Contralateral vs. Control Ipsilateral	0,01413	>0,9999
Control Contralateral vs. CFA Contralateral	0,1311	0,9997
Control Contralateral vs. CFA Ipsilateral	10,63	<0,0001
Control Ipsilateral vs. CFA Contralateral	0,1447	0,9996
Control Ipsilateral vs. CFA Ipsilateral	10,62	<0,0001
CFA Contralateral vs. CFA Ipsilateral	10,42	<0,0001

Data is shown on Figure 3B.

Supplementary Table S7.

Statistical analysis of the *in vivo* bioluminescent data detecting macrophage NADPH oxidase activity in the animals with chronic CFA treatment.

**Macrophage NADPH oxidase activity**		
**Two-way ANOVA**		
**Main effects**	***F* (DFn, DFd) value**	***P* value**
**Contralateral *versus* ipsilateral**	*F* (1, 34) = 17,16	0,0002
**CFA treatment effect**	*F* (1, 34) = 16,34	0,0003
**Interaction**	*F* (1, 34) = 17,6	0,0002
**Tukey's multiple comparisons test**		
**Contralateral *versus* ipsilateral**	***t* value**	***P* value**
Control Contralateral vs. Control Ipsilateral	0,05111	>0,9999
Control Contralateral vs. CFA Contralateral	0,1519	0,9995
Control Contralateral vs. CFA Ipsilateral	8,185	<0,0001
Control Ipsilateral vs. CFA Contralateral	0,09948	0,9999
Control Ipsilateral vs. CFA Ipsilateral	8,238	<0,0001
CFA Contralateral vs. CFA Ipsilateral	8,566	<0,0001

Data is shown on Figure 3B.

Supplementary Table S8.

Statistical analysis of the *in vivo* bioluminescent data detecting the myeloperoxidase enzyme activity of neutrophil granulocytes in the animals of the acute inflammatory phase.

**Neutrophil myeloperoxidase activity**		
**Two-way ANOVA**		
**Main effects**	***F* (DFn, DFd) value**	***P* value**
**Contralateral *versus* ipsilateral**	*F* (1, 84) = 62,79	<0,0001
**CFA treatment effect**	*F* (1, 84) = 43,88	<0,0001
**Interaction**	*F* (1, 84) = 45,32	<0,0001
**Tukey's multiple comparisons test**		
**Contralateral *versus* ipsilateral**	***t* value**	***P* value**
Control Contralateral vs. Control Ipsilateral	1,249	0,8134
Control Contralateral vs. CFA Contralateral	0,107	0,9998
Control Contralateral vs. CFA Ipsilateral	14,41	<0,0001
Control Ipsilateral vs. CFA Contralateral	1,312	0,7899
Control Ipsilateral vs. CFA Ipsilateral	13,48	<0,0001
CFA Contralateral vs. CFA Ipsilateral	14,04	<0,0001

Data is shown on Figure 3D.

Supplementary Table S9.

Statistical analysis of the *in vivo* bioluminescent data detecting the myeloperoxidase enzyme activity of neutrophil granulocytes in the animals of the chronic inflammatory phase.

**Neutrophil myeloperoxidase activity**		
**Two-way ANOVA**		
**Main effects**	***F* (DFn, DFd) value**	***P* value**
**Contralateral *versus* ipsilateral**	*F* (1, 36) = 13.47	0,0008
**CFA treatment effect**	*F* (1, 36) = 8.231	0,0068
**Interaction**	*F* (1, 36) = 8.387	0,0064
**Tukey's multiple comparisons test**		
**Contralateral *versus* ipsilateral**	***t* value**	***P* value**
Control Contralateral vs. Control Ipsilateral	0,7736	0,9468
Control Contralateral vs. CFA Contralateral	0,02704	>0,9999
Control Contralateral vs. CFA Ipsilateral	6,539	0,0003
Control Ipsilateral vs. CFA Contralateral	0,8006	0,9415
Control Ipsilateral vs. CFA Ipsilateral	5,765	0,0013
CFA Contralateral vs. CFA Ipsilateral	6,566	0,0003

Data is shown on Figure 3D.

Supplementary Table S10.

Statistical analysis of the red blood cell count data.

Red blood cell count		
**Two-way ANOVA**		
**Main effects**	***F* (DFn, DFd) value**	***P* value**
**Time effect**	*F* (1, 22) = 2,723	0,1131
**CFA treatment effect**	*F* (1, 22) = 1,665	0,2103
**Interaction: Time × CFA treatment**	*F* (1, 22) = 0,04935	0,8263
**Tukey's multiple comparisons test**		
**Control *versus* CFA**	***t* value**	***P* value**
Acute Control vs. Acute CFA	1,363	0,7709
Acute Control vs. Chronic Control	1,428	0,7455
Acute Control vs. Chronic CFA	0,3598	0,9941
Acute CFA vs. Chronic Control	2,94	0,191
Acute CFA vs. Chronic CFA	1,872	0,5581
Chronic Control vs. Chronic CFA	1,218	0,8245

Data is shown on Figure 4A.

Supplementary Table S11.

Statistical analysis of the white blood cell count data.

White blood cell count		
**Two-way ANOVA**		
**Main effects**	***F* (DFn, DFd) value**	***P* value**
**Time effect**	*F* (1, 22) = 0,0535	0,8192
**CFA treatment effect**	*F* (1, 22) = 2,899	0,1027
**Interaction: Time × CFA treatment**	*F* (1, 22) = 1,779	0,1959
**Tukey's multiple comparisons test**		
**Control *versus* CFA**	***t* value**	***P* value**
Acute :Control vs. Acute CFA	2,737	0,2427
Acute Control vs. Chronic Control	1,102	0,8629
Acute Control vs. Chronic CFA	1,471	0,728
Acute CFA vs. Chronic Control	1,934	0,5319
Acute CFA vs. Chronic CFA	1,565	0,6893
Chronic Control vs. Chronic CFA	0,4207	0,9906

Data is shown on Figure 4B.

Supplementary Table S12.

Statistical analysis of the platelet count data.

Platelet count		
**Two-way ANOVA**		
**Main effects**	***F* (DFn, DFd) value**	***P* value**
**Time effect**	*F* (1, 22) = 5,787	0,025
**CFA treatment effect**	*F* (1, 22) = 0,01797	0,8946
**Interaction: Time × CFA treatment**	*F* (1, 22) = 5,076	0,0346
**Tukey's multiple comparisons test**		
**Control *versus* CFA**	***t* value**	***P* value**
Acute Control vs. Acute CFA	1,91	0,5421
Acute Control vs. Chronic Control	0,1525	0,9995
Acute Control vs. Chronic CFA	2,54	0,3019
Acute CFA vs. Chronic Control	2,271	0,3958
Acute CFA vs. Chronic CFA	4,659	0,0162
Chronic Control vs. Chronic CFA	2,722	0,247

Data is shown on Figure 4C.

Supplementary Table S13.

Statistical analysis of the lymphocyte absolute number data.

Lymphocyte (absolute number)		
**Two-way ANOVA**		
**Main effects**	***F* (DFn, DFd) value**	***P* value**
**Time effect**	F (1, 22) = 0,1114	P=0,7414
**CFA treatment effect**	F (1, 22) = 2,109	P=0,1589
**Interaction: Time × CFA treatment**	F (1, 22) = 5,917	P=0,0225
**Tukey's multiple comparisons test**		
**Control *versus* CFA**	***t* value**	***P* value**
Acute Control vs. Acute CFA	3,654	0,0712
Acute Control vs. Chronic Control	2,251	0,4014
Acute Control vs. Chronic CFA	1,199	0,831
Acute CFA vs. Chronic Control	1,68	0,64
Acute CFA vs. Chronic CFA	2,602	0,2792
Chronic Control vs. Chronic CFA	1,051	0,8786

Data is shown on Figure 4D.

Supplementary Table S14.

Statistical analysis of the lymphocyte percentage data.

Lymphocyte %		
**Two-way ANOVA**		
**Main effects**	***F* (DFn, DFd) value**	***P* value**
**Time effect**	*F* (1, 22) = 0,005079	0,9438
**CFA treatment effect**	*F* (1, 22) = 0,3919	0,5377
**Interaction: Time × CFA treatment**	*F* (1, 22) = 4,744	0,0404
**Tukey's multiple comparisons test**		
**Control *versus* CFA**	***t* value**	***P* value**
Acute Control vs. Acute CFA	2,528	0,3058
Acute Control vs. Chronic Control	2,249	0,4042
Acute Control vs. Chronic CFA	0,6973	0,9598
Acute CFA vs. Chronic Control	0,5548	0,979
Acute CFA vs. Chronic CFA	2,107	0,4603
Chronic Control vs. Chronic CFA	1,77	0,6022

Data is shown on Figure 4D.

Supplementary Table S15.

Statistical analysis of the neutrophil granulocyte absolute number data.

Neutrophil granulocyte (absolute number)		
**Two-way ANOVA**		
**Main effects**	***F* (DFn, DFd) value**	***P* value**
**Time effect**	F (1, 22) = 0,04947	0,8258
**CFA treatment effect**	F (1, 22) = 0,02976	0,8644
**Interaction: Time × CFA treatment**	F (1, 22) = 3,785	0,063
**Tukey's multiple comparisons test**		
**Control *versus* CFA**	***t* value**	***P* value**
Acute Control vs. Acute CFA	1,668	0,6452
Acute Control vs. Chronic Control	1,848	0,5674
Acute Control vs. Chronic CFA	0,4235	0,9904
Acute CFA vs. Chronic Control	0,04694	>0,9999
Acute CFA vs. Chronic CFA	2,039	0,4862
Chronic Control vs. Chronic CFA	2,271	0,3935

Data is shown on Figure 4E.

Supplementary Table S16.

Statistical analysis of the neutrophil granulocyte percentage data.

Neutrophil granulocyte %		
**Two-way ANOVA**		
**Main effects**	***F* (DFn, DFd) value**	***P* value**
**Time effect**	*F* (1, 22) = 0,713	0,4076
**CFA treatment effect**	*F* (1, 22) = 1,789	0,1947
**Interaction: Time × CFA treatment**	*F* (1, 22) = 9,147	0,0062
**Tukey's multiple comparisons test**		
**Control *versus* CFA**	***t* value**	***P* value**
Acute Control vs. Acute CFA	3,932	0,0497
Acute Control vs. Chronic Control	2,18	0,431
Acute Control vs. Chronic CFA	0,4931	0,9851
Acute CFA vs. Chronic Control	2,182	0,4303
Acute CFA vs. Chronic CFA	3,869	0,0545
Chronic Control vs. Chronic CFA	1,923	0,5364

Data is shown on Figure 4E.

Supplementary Table S17.

Statistical analysis of the monocyte absolute number data.

Monocyte (absolute number)		
**Two-way ANOVA**		
**Main effects**	***F* (DFn, DFd) value**	***P* value**
**Time effect**	F (1, 22) = 3,406	0,0768
**CFA treatment effect**	F (1, 22) = 0,9606	0,3364
**Interaction: Time × CFA treatment**	F (1, 22) = 7,933	0,0093
**Tukey's multiple comparisons test**		
**Control *versus* CFA**	***t* value**	***P* value**
Acute Control vs. Acute CFA	3,571	0,0802
Acute Control vs. Chronic Control	1,041	0,8815
Acute Control vs. Chronic CFA	0,928	0,9124
Acute CFA vs. Chronic Control	2,658	0,2622
Acute CFA vs. Chronic CFA	4,385	0,023
Chronic Control vs. Chronic CFA	1,969	0,5155

Data is shown on Figure 4F.

Supplementary Table S18.

Statistical analysis of the monocyte percentage data.

**Monocyte %**		
**Two-way ANOVA**		
**Main effects**	***F* (DFn, DFd) value**	***P* value**
**Time effect**	*F* (1, 22) = 4,471	0,046
**CFA treatment effect**	*F* (1, 22) = 0,4063	0,5304
**Interaction: Time × CFA treatment**	*F* (1, 22) = 7,752	0,0108
**Tukey's multiple comparisons test**		
**Control *versus* CFA**	***t* value**	***P* value**
Acute Control vs. Acute CFA	3,084	0,1598
Acute Control vs. Chronic Control	0,6699	0,9641
Acute Control vs. Chronic CFA	1,477	0,7257
Acute CFA vs. Chronic Control	2,752	0,2386
Acute CFA vs. Chronic CFA	4,899	0,011
Chronic Control vs. Chronic CFA	2,448	0,3324

Data is shown on Figure 4F.

Supplementary Table S19.

Statistical analysis of the eosinophil granulocyte absolute number data.

**Eosinophil granulocyte (absolute number)**		
**Two-way ANOVA**		
**Main effects**	***F* (DFn, DFd) value**	***P* value**
**Time effect**	F (1, 22) = 0,6294	0,4351
**CFA treatment effect**	F (1, 22) = 9,569	0,0048
**Interaction: Time × CFA treatment**	F (1, 22) = 1,786	0,1935
**Tukey's multiple comparisons test**		
**Control *versus* CFA**	***t* value**	***P* value**
Acute Control vs. Acute CFA	4,166	0,0326
Acute Control vs. Chronic Control	2,284	0,3888
Acute Control vs. Chronic CFA	4,168	0,0325
Acute CFA vs. Chronic Control	2,163	0,4356
Acute CFA vs. Chronic CFA	0,5108	0,9835
Chronic Control vs. Chronic CFA	1,884	0,5518

Data is shown on Figure 4G.

Supplementary Table S20.

Statistical analysis of the eosinophil granulocyte percentage data.

Eosinophil granulocyte %		
**Two-way ANOVA**		
**Main effects**	***F* (DFn, DFd) value**	***P* value**
**Time effect**	*F* (1, 22) = 0,1548	0,6978
**CFA treatment effect**	*F* (1, 22) = 6,195	0,0209
**Interaction: Time × CFA treatment**	*F* (1, 22) = 0,05384	0,8186
**Tukey's multiple comparisons test**		
**Control *versus* CFA**	***t* value**	***P* value**
Acute Control vs. Acute CFA	2,453	0,3306
Acute Control vs. Chronic Control	0,6255	0,9704
Acute Control vs. Chronic CFA	2,883	0,2048
Acute CFA vs. Chronic Control	2,096	0,4648
Acute CFA vs. Chronic CFA	0,1614	0,9995
Chronic Control vs. Chronic CFA	2,573	0,2911

Data is shown on Figure 4G.

Supplementary Table S21.

Statistical analysis of the basophil granulocyte absolute number data.

Basophil granulocyte (absolute number)		
**Two-way ANOVA**		
**Main effects**	***F* (DFn, DFd) value**	***P* value**
**Time effect**	F (1, 22) = 0,08002	0,7796
**CFA treatment effect**	F (1, 22) = 4,783	0,0383
**Interaction: Time × CFA treatment**	F (1, 22) = 0,06717	0,7976
**Tukey's multiple comparisons test**		
**Control *versus* CFA**	***t* value**	***P* value**
Acute Control vs. Acute CFA	2,301	0,3824
Acute Control vs. Chronic Control	0,02542	>0,9999
Acute Control vs. Chronic CFA	2,042	0,485
Acute CFA vs. Chronic Control	2,323	0,3741
Acute CFA vs. Chronic CFA	0,5098	0,9836
Chronic Control vs. Chronic CFA	2,067	0,4745

Data is shown on Figure 4H.

Supplementary Table S22.

Statistical analysis of the basophil granulocyte percentage data.

Basophil granulocyte %		
**Two-way ANOVA**		
**Main effects**	***F* (DFn, DFd) value**	***P* value**
**Time effect**	*F* (1, 22) = 0,0425	0,4156
**CFA treatment effect**	*F* (1, 22) = 6,883	0,0473
**Interaction: Time × CFA treatment**	*F* (1, 22) = 4,414	0,8386
**Tukey's multiple comparisons test**		
**Control *versus* CFA**	***t* value**	***P* value**
Acute Control vs. Acute CFA	1,708	0,6286
Acute Control vs. Chronic Control	1,036	0,883
Acute Control vs. Chronic CFA	1,271	0,8055
Acute CFA vs. Chronic Control	2,931	0,1933
Acute CFA vs. Chronic CFA	0,6235	0,9707
Chronic Control vs. Chronic CFA	2,63	0,2736

Data is shown on Figure 4H.

Supplementary Table S23.

Statistical analysis of the BrdU-positive cell number data.

BrdU-positive cell numbers		
**Two-way ANOVA**		
**Main effects**	***F* (DFn, DFd) value**	***P* value**
**Time effect**	*F* (1, 30) = 17,91	0,0002
**CFA treatment effect**	*F* (1, 30) = 0,7392	0,3967
**Interaction:<br>Time × CFA treatment**	*F* (1, 30) = 1,095	0,3036
**Tukey's multiple comparisons test**		
**Control *versus* CFA**	***t* value**	***P* value**
Acute Control *vs*. Acute CFA	0,1816	0,9992
Acute Control *vs*. Chronic Control	3,185	0,1324
Acute Control *vs*. Chronic CFA	5,091	0,0059
Acute CFA *vs*. Chronic Control	3,372	0,102
Acute CFA *vs*. Chronic CFA	5,278	0,0042
Chronic Control *vs*. Chronic CFA	1,965	0,5155

Data is shown on Figure 5B.

Supplementary Table S24.

Statistical analysis of the doublecortin-positive cell number data.

DCX-positive cell numbers in the dentate gyrus		
**Two-way ANOVA**		
**Main effects**	***F* (DFn, DFd) value**	***P* value**
**Time effect**	*F* (1, 30) = 13,82	0,0008
**CFA treatment effect**	*F* (1, 30) = 17,89	0,0002
**Interaction: Time × CFA treatment**	*F* (1, 30) = 1,16	0,29
**Tukey's multiple comparisons test**		
**Control *versus* CFA**	***t* value**	***P* value**
Acute Control *vs*. Acute CFA	3,063	0,156
Acute Control *vs*. Chronic Control	2,64	0,2636
Acute Control *vs*. Chronic CFA	7,946	<0,0001
Acute CFA *vs*. Chronic Control	0,5123	0,9834
Acute CFA *vs*. Chronic CFA	4,794	0,0101
Chronic Control *vs*. Chronic CFA	5,47	0,0029

Data is shown on Figure 6B.

Supplementary Table S25.

Statistical analysis of the Iba1-positive cell number data.

Iba1-positive microglia cell density in the dentate gyrus		
**Two-way ANOVA**		
**Main effects**	***F* (DFn, DFd) value**	***P* value**
**Time effect**	*F* (1, 27) = 0,8227	0,3724
**CFA treatment effect**	*F* (1, 27) = 0,001398	0,9704
**Interaction: Time × CFA treatment**	*F* (1, 27) = 0,05987	0,8085
**Tukey's multiple comparisons test**		
**Control *versus* CFA**	***t* value**	***P* value**
Acute Control *vs*. Acute CFA	0,2784	0,9972
Acute Control *vs*. Chronic Control	0,6329	0,9695
Acute Control *vs*. Chronic CFA	0,8814	0,9237
Acute CFA *vs*. Chronic Control	0,9321	0,9114
Acute CFA *vs*. Chronic CFA	1,211	0,8271
Chronic Control *vs*. Chronic CFA	0,2101	0,9988

Data is shown on Figure 7B.

Supplementary Table S26.

Statistical analysis of the CD68-positive microglia / macrophages cell number data.

CD68-positive microglia cell density in the dentate gyrus		
**Two-way ANOVA**		
**Main effects**	***F* (DFn, DFd) value**	***P* value**
**Time effect**	*F* (1, 23) = 8,397	0,0081
**CFA treatment effect**	*F* (1, 23) = 39,68	<0,0001
**Interaction: Time × CFA treatment**	*F* (1, 23) = 2,721	0,1126
**Tukey's multiple comparisons test**		
**Control *versus* CFA**	***t* value**	***P* value**
Acute Control *vs*. Acute CFA	7,834	<0,0001
Acute Control *vs*. Chronic Control	4,481	0,0208
Acute Control *vs.* Chronic CFA	9,743	<0,0001
Acute CFA *vs*. Chronic Control	3,23	0,1312
Acute CFA *vs*. Chronic CFA	1,267	0,8069
Chronic Control *vs*. Chronic CFA	4,721	0,0141

Data is shown on Figure 8B.

Supplementary Table S27.

Statistical analysis of the IL-1α cytokine concentration data in the hind paws.

**Ankle IL-1α**		
**Two-way ANOVA**		
**Main effects**	***F* (DFn, DFd) value**	***P* value**
**Time effect**	F (1, 20) = 1,254	0,2761
**CFA treatment effect**	F (1, 20) = 7,608	0,0121
**Interaction: Time × CFA treatment**	F (1, 20) = 8,290	0,0093
**Tukey's multiple comparisons test**		
**Control *versus* CFA**	***t* value**	***P* value**
Acute Control vs. Acute CFA	5,637	0,0037
Acute Control vs. Chronic Control	1,759	0,6073
Acute Control vs. Chronic CFA	1,638	0,6588
Acute CFA vs. Chronic Control	3,878	0,0561
Acute CFA vs. Chronic CFA	3,999	0,0471
Chronic Control vs. Chronic CFA	0,121	0,9998

Data is shown on Figure 9A.

Supplementary Table S28.

Statistical analysis of the IL-1α cytokine concentration data in the peripheral blood.

**Blood IL-1α**		
**Two-way ANOVA**		
**Main effects**	***F* (DFn, DFd) value**	***P* value**
**Time effect**	F (1, 20) = 3,350	0,0821
**CFA treatment effect**	F (1, 20) = 0,1250	0,7274
**Interaction: Time × CFA treatment**	F (1, 20) = 1,007	0,3275
**Tukey's multiple comparisons test**		
**Control *versus* CFA**	***t* value**	***P* value**
Acute Control vs. Acute CFA	0,6501	0,9669
Acute Control vs. Chronic Control	2,834	0,2197
Acute Control vs. Chronic CFA	1,477	0,726
Acute CFA vs. Chronic Control	2,184	0,4313
Acute CFA vs. Chronic CFA	0,8267	0,9356
Chronic Control vs. Chronic CFA	1,357	0,7733

Data is shown on Figure 9A.

Supplementary Table S29.

Statistical analysis of the IL-1α cytokine concentration data in the hippocampus.

**Hippocampus IL-1α**		
**Two-way ANOVA**		
**Main effects**	***F*** **(DFn, DFd) value**	***P*** **value**
**Time effect**	F (1, 20) = 92,11	<0,0001
**CFA treatment effect**	F (1, 20) = 1,105	0,3058
**Interaction: Time × CFA treatment**	F (1, 20) = 0,1289	0,7233
**Tukey's multiple comparisons test**		
**Control** ***versus*** **CFA**	***t*** **value**	***P*** **value**
Acute Control vs. Acute CFA	0,6919	0,9606
Acute Control vs. Chronic Control	9,238	<0,0001
Acute Control vs. Chronic CFA	10,65	<0,0001
Acute CFA vs. Chronic Control	8,546	<0,0001
Acute CFA vs. Chronic CFA	9,956	<0,0001
Chronic Control vs. Chronic CFA	1,41	0,7527

Data is shown on Figure 9A.

Supplementary Table S30.

Statistical analysis of the IL-4 cytokine concentration data in the hind paw.

**Ankle IL-4**		
**Two-way ANOVA**		
**Main effects**	***F*** **(DFn, DFd) value**	***P*** **value**
**Time effect**	F (1, 20) = 4,953	0,0377
**CFA treatment effect**	F (1, 20) = 4,953	0,0377
**Interaction: Time × CFA treatment**	F (1, 20) = 4,953	0,0377
**Tukey's multiple comparisons test**		
**Control** ***versus*** **CFA**	***t*** **value**	***P*** **value**
Acute Control vs. Acute CFA	4,451	0,0241
Acute Control vs. Chronic Control	0	>0,9999
Acute Control vs. Chronic CFA	0	>0,9999
Acute CFA vs. Chronic Control	4,451	0,0241
Acute CFA vs. Chronic CFA	4,451	0,0241
Chronic Control vs. Chronic CFA	0	>0,9999

Data is shown on Figure 9B.

Supplementary Table S31.

Statistical analysis of the IL-4 cytokine concentration data in the peripheral blood.

**Blood IL-4**		
**Two-way ANOVA**		
**Main effects**	***F* (DFn, DFd) value**	***P* value**
**Time effect**	F (1, 20) = 1,000	0,3293
**CFA treatment effect**	F (1, 20) = 1,000	0,3293
**Interaction: Time × CFA treatment**	F (1, 20) = 1,000	0,3293
**Tukey's multiple comparisons test**		
**Control *versus* CFA**	***t* value**	***P* value**
Acute Control vs. Acute CFA	2	0,5054
Acute Control vs. Chronic Control	0	>0,9999
Acute Control vs. Chronic CFA	0	>0,9999
Acute CFA vs. Chronic Control	2	0,5054
Acute CFA vs. Chronic CFA	2	0,5054
Chronic Control vs. Chronic CFA	0	>0,9999

Data is shown on Figure 9B.

Supplementary Table S32.

Statistical analysis of the IL-6 cytokine concentration data in the hind paws.

**Ankle IL-6**		
**Two-way ANOVA**		
**Main effects**	***F* (DFn, DFd) value**	***P* value**
**Time effect**	F (1, 20) = 17,96	0,0004
**CFA treatment effect**	F (1, 20) = 19,69	0,0003
**Interaction: Time × CFA treatment**	F (1, 20) = 14,07	0,0013
**Tukey's multiple comparisons test**		
**Control *versus* CFA**	***t* value**	***P* value**
Acute Control vs. Acute CFA	8,189	<0,0001
Acute Control vs. Chronic Control	0,4868	0,9856
Acute Control vs. Chronic CFA	0,1994	0,999
Acute CFA vs. Chronic Control	8,676	<0,0001
Acute CFA vs. Chronic CFA	7,99	<0,0001
Chronic Control vs. Chronic CFA	0,6862	0,9615

Data is shown on Figure 9C.

Supplementary Table S33.

Statistical analysis of the IL-6 cytokine concentration data in the peripheral blood.

**Blood IL-6**		
**Two-way ANOVA**		
**Main effects**	***F*** **(DFn, DFd) value**	***P*** **value**
**Time effect**	F (1, 20) = 6,329	0,0205
**CFA treatment effect**	F (1, 20) = 8,989	0,0071
**Interaction: Time × CFA treatment**	F (1, 20) = 2,747	0,113
**Tukey's multiple comparisons test**		
**Control *versus* CFA**	***t*** **value**	***P*** **value**
Acute Control vs. Acute CFA	4,656	0,0176
Acute Control vs. Chronic Control	0,8583	0,9287
Acute Control vs. Chronic CFA	0,4825	0,9859
Acute CFA vs. Chronic Control	5,514	0,0045
Acute CFA vs. Chronic CFA	4,173	0,0365
Chronic Control vs. Chronic CFA	1,341	0,7796

Data is shown on Figure 9C.

Supplementary Table S34.

Statistical analysis of the IL-10 cytokine concentration data in the hind paws.

**Ankle IL-10**		
**Two-way ANOVA**		
**Main effects**	***F*** **(DFn, DFd) value**	***P*** **value**
**Time effect**	F (1, 20) = 2,353	0,1407
**CFA treatment effect**	F (1, 20) = 0,6596	0,4263
**Interaction: Time × CFA treatment**	F (1, 20) = 4,176	0,0544
**Tukey's multiple comparisons test**		
**Control *versus* CFA**	***t*** **value**	***P*** **value**
Acute Control vs. Acute CFA	2,856	0,2143
Acute Control vs. Chronic Control	3,578	0,0855
Acute Control vs. Chronic CFA	2,346	0,3703
Acute CFA vs. Chronic Control	0,7219	0,9556
Acute CFA vs. Chronic CFA	0,5096	0,9835
Chronic Control vs. Chronic CFA	1,231	0,8197

Data is shown on Figure 9D.

Supplementary Table S35.

Statistical analysis of the IL-10 cytokine concentration data in the peripheral blood.

**Blood IL-10**		
**Two-way ANOVA**		
**Main effects**	***F* (DFn, DFd) value**	***P* value**
**Time effect**	F (1, 20) = 1,522	0,2316
**CFA treatment effect**	F (1, 20) = 0,6100	0,4439
**Interaction: Time × CFA treatment**	F (1, 20) = 4,694	0,0425
**Tukey's multiple comparisons test**		
**Control *versus* CFA**	***t* value**	***P* value**
Acute Control vs. Acute CFA	2,948	0,1922
Acute Control vs. Chronic Control	3,4	0,1085
Acute Control vs. Chronic CFA	2,015	0,4994
Acute CFA vs. Chronic Control	0,4526	0,9883
Acute CFA vs. Chronic CFA	0,9329	0,9109
Chronic Control vs. Chronic CFA	1,386	0,7624

Data is shown on Figure 9D.

Supplementary Table S36.

Statistical analysis of the IL-10 cytokine concentration data in the hippocampus.

**Hippocampus IL-10**		
**Two-way ANOVA**		
**Main effects**	***F* (DFn, DFd) value**	***P* value**
**Time effect**	F (1, 20) = 12,60	0,002
**CFA treatment effect**	F (1, 20) = 0,06992	0,7942
**Interaction: Time × CFA treatment**	F (1, 20) = 0,1157	0,7373
**Tukey's multiple comparisons test**		
**Control *versus* CFA**	***t* value**	***P* value**
Acute Control vs. Acute CFA	0,07573	>0,9999
Acute Control vs. Chronic Control	3,209	0,1392
Acute Control vs. Chronic CFA	3,814	0,0615
Acute CFA vs. Chronic Control	3,285	0,1263
Acute CFA vs. Chronic CFA	3,889	0,0552
Chronic Control vs. Chronic CFA	0,6046	0,9731

Data is shown on Figure 9D.

Supplementary Table S37.

Statistical analysis of the KC concentration data in the hind paws.

Ankle KC		
**Two-way ANOVA**		
**Main effects**	***F* (DFn, DFd) value**	***P* value**
**Time effect**	P=0,1045	0,1045
**CFA treatment effect**	P<0,0001	<0,0001
**Interaction: Time × CFA treatment**	P=0,0848	0,0848
**Tukey's multiple comparisons test**		
**Control *versus* CFA**	***t* value**	***P* value**
Acute Control vs. Acute CFA	9,048	<0,0001
Acute Control vs. Chronic Control	0,1128	0,9998
Acute Control vs. Chronic CFA	5,534	0,0044
Acute CFA vs. Chronic Control	8,935	<0,0001
Acute CFA vs. Chronic CFA	3,514	0,0932
Chronic Control vs. Chronic CFA	5,421	0,0053

Data is shown on Figure 10A.

Supplementary Table S38.

Statistical analysis of the KC concentration data in the peripheral blood.

Blood KC		
**Two-way ANOVA**		
**Main effects**	***F* (DFn, DFd) value**	***P* value**
**Time effect**	F (1, 20) = 0,6868	0,417
**CFA treatment effect**	F (1, 20) = 3,424	0,0791
**Interaction: Time × CFA treatment**	F (1, 20) = 1,051	0,3175
**Tukey's multiple comparisons test**		
**Control *versus* CFA**	***t* value**	***P* value**
Acute Control vs. Acute CFA	0,825	0,9359
Acute Control vs. Chronic Control	0,1965	0,999
Acute Control vs. Chronic CFA	2,679	0,2619
Acute CFA vs. Chronic Control	1,022	0,887
Acute CFA vs. Chronic CFA	1,854	0,5669
Chronic Control vs. Chronic CFA	2,876	0,2094

Data is shown on Figure 10A.

Supplementary Table S39.

Statistical analysis of the KC concentration data in the hippocampus.

Hippocampus KC		
**Two-way ANOVA**		
**Main effects**	***F* (DFn, DFd) value**	***P* value**
**Time effect**	P<0,0001	<0,0001
**CFA treatment effect**	P=0,1060	0,106
**Interaction: Time × CFA treatment**	P=0,0521	0,0521
**Tukey's multiple comparisons test**		
**Control *versus* CFA**	***t* value**	***P* value**
Acute Control vs. Acute CFA	0,3723	0,9934
Acute Control vs. Chronic Control	8,166	<0,0001
Acute Control vs. Chronic CFA	4,408	0,0257
Acute CFA vs. Chronic Control	7,794	0,0001
Acute CFA vs. Chronic CFA	4,036	0,0447
Chronic Control vs. Chronic CFA	3,758	0,0665

Data is shown on Figure 10A.

Supplementary Table S40.

Statistical analysis of the MIP-2 concentration data in the hind paws.

Ankle MIP-2		
**Two-way ANOVA**		
**Main effects**	***F* (DFn, DFd) value**	***P* value**
**Time effect**	F (1, 20) = 4,550	0,0455
**CFA treatment effect**	F (1, 20) = 19,43	0,0003
**Interaction: Time × CFA treatment**	F (1, 20) = 4,308	0,0511
**Tukey's multiple comparisons test**		
**Control *versus* CFA**	***t* value**	***P* value**
Acute Control vs. Acute CFA	6,484	0,001
Acute Control vs. Chronic Control	0,05763	>0,9999
Acute Control vs. Chronic CFA	2,275	0,3964
Acute CFA vs. Chronic Control	6,541	0,0009
Acute CFA vs. Chronic CFA	4,209	0,0346
Chronic Control vs. Chronic CFA	2,333	0,3751

Data is shown on Figure 10B.

Supplementary Table S41.

Statistical analysis of the MIP-2 concentration data in the peripheral blood.

Blood MIP-2		
**Two-way ANOVA**		
**Main effects**	***F* (DFn, DFd) value**	***P* value**
**Time effect**	F (1, 20) = 0,1105	0,7431
**CFA treatment effect**	F (1, 20) = 0,2816	0,6015
**Interaction: Time × CFA treatment**	F (1, 20) = 3,329	0,083
**Tukey's multiple comparisons test**		
**Control *versus* CFA**	***t* value**	***P* value**
Acute Control vs. Acute CFA	1,294	0,7971
Acute Control vs. Chronic Control	2,157	0,4418
Acute Control vs. Chronic CFA	0,1983	0,999
Acute CFA vs. Chronic Control	0,863	0,9276
Acute CFA vs. Chronic CFA	1,492	0,7197
Chronic Control vs. Chronic CFA	2,355	0,367

Data is shown on Figure 10B.

Supplementary Table S42.

Statistical analysis of the MIP-2 concentration data in the hippocampus.

Hippocampus MIP-2		
**Two-way ANOVA**		
**Main effects**	***F* (DFn, DFd) value**	***P* value**
**Time effect**	F (1, 20) = 26,05	<0,0001
**CFA treatment effect**	F (1, 20) = 0,3319	0,571
**Interaction: Time × CFA treatment**	F (1, 20) = 1,795	0,1954
**Tukey's multiple comparisons test**		
**Control *versus* CFA**	***t* value**	***P* value**
Acute Control vs. Acute CFA	1,916	0,5407
Acute Control vs. Chronic Control	3,764	0,066
Acute Control vs. Chronic CFA	4,527	0,0214
Acute CFA vs. Chronic Control	5,68	0,0035
Acute CFA vs. Chronic CFA	6,443	0,001
Chronic Control vs. Chronic CFA	0,7636	0,9482

Data is shown on Figure 10B.

Supplementary Table S43.

Statistical analysis of the TNF-α concentration data in the hind paws.

Ankle TNF-a		
**Two-way ANOVA**		
**Main effects**	***F* (DFn, DFd) value**	***P* value**
**Time effect**	F (1, 20) = 0,7646	0,3923
**CFA treatment effect**	F (1, 20) = 65,64	<0,0001
**Interaction: Time × CFA treatment**	F (1, 20) = 0,7646	0,3923
**Tukey's multiple comparisons test**		
**Control *versus* CFA**	***t* value**	***P* value**
Acute Control vs. Acute CFA	8,976	<0,0001
Acute Control vs. Chronic Control	0	>0,9999
Acute Control vs. Chronic CFA	7,227	0,0003
Acute CFA vs. Chronic Control	8,976	<0,0001
Acute CFA vs. Chronic CFA	1,749	0,6118
Chronic Control vs. Chronic CFA	7,227	0,0003

Data is shown on Figure 10C.

Supplementary Table S44.

Statistical analysis of the TNF-α concentration data in the peripheral blood.

Blood TNF-a		
**Two-way ANOVA**		
**Main effects**	***F* (DFn, DFd) value**	***P* value**
**Time effect**	F (1, 20) = 1,889	0,1845
**CFA treatment effect**	F (1, 20) = 0,1105	0,743
**Interaction: Time × CFA treatment**	F (1, 20) = 0,1105	0,743
**Tukey's multiple comparisons test**		
**Control *versus* CFA**	***t* value**	***P* value**
Acute Control vs. Acute CFA	0	>0,9999
Acute Control vs. Chronic Control	1,042	0,8811
Acute Control vs. Chronic CFA	1,707	0,6296
Acute CFA vs. Chronic Control	1,042	0,8811
Acute CFA vs. Chronic CFA	1,707	0,6296
Chronic Control vs. Chronic CFA	0,6649	0,9648

Data is shown on Figure 10C.

Supplementary Table S45.

Statistical analysis of the TNF-α concentration data in the hippocampus.

Hippocampus TNF-a		
Two-way ANOVA		
**Main effects**	***F* (DFn, DFd) value**	***P* value**
**Time effect**	F (1, 20) = 1,000	0,3293
**CFA treatment effect**	F (1, 20) = 1,000	0,3293
**Interaction: Time × CFA treatment**	F (1, 20) = 1,000	0,3293
**Tukey's multiple comparisons test**		
**Control *versus* CFA**	***t* value**	***P* value**
Acute Control vs. Acute CFA	0	>0,9999
Acute Control vs. Chronic Control	0	>0,9999
Acute Control vs. Chronic CFA	2	0,5054
Acute CFA vs. Chronic Control	0	>0,9999
Acute CFA vs. Chronic CFA	2	0,5054
Chronic Control vs. Chronic CFA	2	0,5054

Data is shown on Figure 10C.

Supplementary Table S46.

Statistical analysis of the protein concentration data in the hind paws.

Ankle protein concentration		
Two-way ANOVA		
**Main effects**	***F* (DFn, DFd) value**	***P* value**
**Time effect**	F (1, 20) = 27,92	<0,0001
**CFA treatment effect**	F (1, 20) = 384,4	<0,0001
**Interaction: Time × CFA treatment**	F (1, 20) = 69,06	<0,0001
**Tukey's multiple comparisons test**		
**Control *versus* CFA**	***t* value**	***P* value**
Acute Control vs. Acute CFA	27,92	<0,0001
Acute Control vs. Chronic Control	3,026	0,1748
Acute Control vs. Chronic CFA	14,32	<0,0001
Acute CFA vs. Chronic Control	24,89	<0,0001
Acute CFA vs. Chronic CFA	13,59	<0,0001
Chronic Control vs. Chronic CFA	11,3	<0,0001

Data is shown on Figure 10D.

Supplementary Table S47.

Statistical analysis of the protein concentration data in the peripheral blood.

Blood Protein concentration		
**Two-way ANOVA**		
**Main effects**	***F* (DFn, DFd) value**	***P* value**
**Time effect**	F (1, 20) = 26,77	<0,0001
**CFA treatment effect**	F (1, 20) = 2,274	0,1472
**Interaction: Time × CFA treatment**	F (1, 20) = 2,062	0,1665
**Tukey's multiple comparisons test**		
**Control *versus* CFA**	***t* value**	***P* value**
Acute Control vs. Acute CFA	0,07203	>0,9999
Acute Control vs. Chronic Control	3,739	0,0684
Acute Control vs. Chronic CFA	6,682	0,0007
Acute CFA vs. Chronic Control	3,666	0,0756
Acute CFA vs. Chronic CFA	6,61	0,0008
Chronic Control vs. Chronic CFA	2,944	0,1931

Data is shown on Figure 10D.

Supplementary Table S48.

Statistical analysis of the protein concentration data in the hippocampus.

Hippocampus Protein concentration		
**Two-way ANOVA**		
**Main effects**	***F* (DFn, DFd) value**	***P* value**
**Time effect**	F (1, 20) = 20,54	0,0002
**CFA treatment effect**	F (1, 20) = 3,860	0,0635
**Interaction: Time × CFA treatment**	F (1, 20) = 0,002110	0,9638
**Tukey's multiple comparisons test**		
**Control *versus* CFA**	***t* value**	***P* value**
Acute Control vs. Acute CFA	2,011	0,501
Acute Control vs. Chronic Control	4,578	0,0198
Acute Control vs. Chronic CFA	6,497	0,0009
Acute CFA vs. Chronic Control	2,567	0,2956
Acute CFA vs. Chronic CFA	4,486	0,0228
Chronic Control vs. Chronic CFA	1,919	0,5394

Data is shown on Figure 10D.
